# Quantum tomography of electrical currents

**DOI:** 10.1038/s41467-019-11369-5

**Published:** 2019-07-29

**Authors:** R. Bisognin, A. Marguerite, B. Roussel, M. Kumar, C. Cabart, C. Chapdelaine, A. Mohammad-Djafari, J.-M. Berroir, E. Bocquillon, B. Plaçais, A. Cavanna, U. Gennser, Y. Jin, P. Degiovanni, G. Fève

**Affiliations:** 1 Laboratoire de Physique de l’ Ecole normale supérieure, ENS, Université PSL, CNRS, Sorbonne Université, Université Paris-Diderot, Sorbonne Paris Cité, Paris, 75005 France; 2 Univ Lyon, Ens de Lyon, Université Claude Bernard Lyon 1, CNRS, Laboratoire de Physique, F-69342 Lyon, France; 3 European Space Agency—Advanced Concepts Team, ESTEC, Keplerlaan 1, 2201 AZ Noordwijk, The Netherlands; 4 Laboratoire des signaux et systèmes, CNRS, Centrale-Supélec—Université Paris-Saclay, Gif-sur-Yvette, F-91190 France; 5 Centre de Nanosciences et de Nanotechnologies (C2N), CNRS, Univ. Paris-Sud, Université Paris-Saclay, 91120 Palaiseau, France

**Keywords:** Quantum information, Quantum Hall, Quantum physics

## Abstract

In quantum nanoelectronics, time-dependent electrical currents are built from few elementary excitations emitted with well-defined wavefunctions. However, despite the realization of sources generating quantized numbers of excitations, and despite the development of the theoretical framework of time-dependent quantum electronics, extracting electron and hole wavefunctions from electrical currents has so far remained out of reach, both at the theoretical and experimental levels. In this work, we demonstrate a quantum tomography protocol which extracts the generated electron and hole wavefunctions and their emission probabilities from any electrical current. It combines two-particle interferometry with signal processing. Using our technique, we extract the wavefunctions generated by trains of Lorentzian pulses carrying one or two electrons. By demonstrating the synthesis and complete characterization of electronic wavefunctions in conductors, this work offers perspectives for quantum information processing with electrical currents and for investigating basic quantum physics in many-body systems.

## Abstract (web summary)

Elementary excitations of electronic devices have potential use in quantum information but control and readout capabilities are not as developed as they are for more mature systems such as photonic qubits. Here the authors develop and demonstrate a tomographic protocol for electron and hole wavefunctions.

## Introduction

In the field of quantum technologies, controlling elementary excitations such as single photons^1^, single atoms^2^, or single ions^3^ is a resource for encoding quantum information as well as a way to develop our understanding of basic quantum physics in complex many-body problems. In quantum electronics, the availability of on-demand single-electron sources^4–7^ offers the possibility to generate time-dependent electrical currents carrying a controlled number of electron and hole excitations of a degenerate electronic fluid. At low temperatures, phase coherence is preserved, such that describing these excitations in terms of well-defined wavefunctions is meaningful. In addition, by implementing electron sources in ballistic low-dimensional conductors^4,7,8^, the elementary excitations can be guided along one-dimensional channels and used as flying qubits^9,10^ carrying information encoded in their quantum state. However, despite the development of a rich experimental toolbox to generate and propagate electronic states in a controlled way, very few tools are currently available to characterize these states. In particular, measuring electron or hole wavefunctions embedded within a quantum electrical current has, so far, been out of reach.

**Fig. 1:**
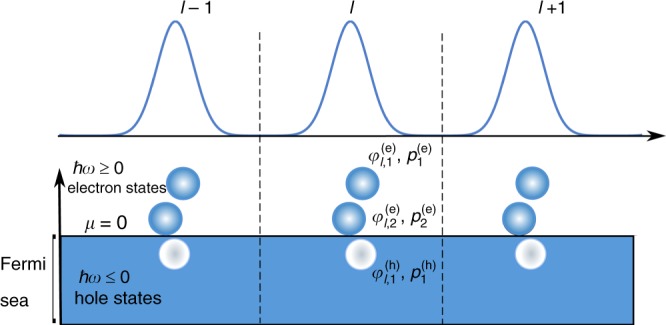
Sketch of the elementary excitations generated by a periodic current *I*(*t*). Electron and hole states are generated at each period above and below the Fermi sea. The time-translated wavefunctions $\varphi _{l,i}^{(\alpha )}(t) = \varphi _i^{(\alpha )}(t - lT)$, for *α = *e (electron) or h (hole) and 1 ≤ *i* ≤ *N*_*α*_ are emitted at each period with probability $p_i^{(\alpha )}$

This absence of a universal tomography protocol in the fermionic case may seem peculiar, considering that such protocols are now commonly implemented to reconstruct the state of bosonic fields^11,12^. However, there are important differences between bosonic and fermionic fields. First, bosonic tomography protocols involve the use of a classical field^12^, which has no counterpart for fermions. Second, the vacuum of a fermionic system being a Fermi sea, the electron and hole excitations are thus defined by the addition and removal of a particle. Quantum-state reconstruction in the fermionic case can be illustrated by the sketch of Fig. 1. In a one-dimensional conductor, a *T*-periodic source generates a time-dependent current consisting of periodic pulses labeled by the index $l \in {\Bbb Z}$. To define unambiguously the electron and hole excitations, we take the conductor at chemical potential *μ*  = 0 and temperature *T*_el_ = 0 K as a reference. The electronic excitations correspond to the filling of the states above the Fermi sea (energy ℏ*ω* ≥ 0) and the hole excitations to the emptying of the states below the Fermi sea (ℏ*ω* ≤ 0). We introduce in Fig. 1 the emitted time-translated electron (e) and hole (h) wavefunctions $\varphi _{l,i}^{(\alpha )}(t) = \varphi _i^{(\alpha )}(t - lT)$, and their emission probabilities $p_i^{(\alpha )},$ where *α* = e or h labels the electron or hole states and *i* runs from 1 to *N*_*α*_, the total number of electron (*N*_e_) and hole (*N*_h_) wavefunctions emitted per period. These emitted electron and hole wavefunctions form a set of mutually orthogonal states: $\langle \varphi _{l{\prime},i{\prime}}^{(\alpha{\prime})}|\varphi _{l,i}^{(\alpha )}\rangle = \delta _{i,i\prime }\,\delta _{\alpha ,\alpha\prime }\,\delta _{l,l\prime }$.

Here, by combining two-particle interferometry^13^ with signal processing^14^, we demonstrate a quantum current analyzer, which extracts the emitted wavefunctions $\varphi _{l,i}^{(\alpha )}(t)$ and their emission probabilities $p_i^{(\alpha )}$ from any periodic electrical current. For benchmarking, we first apply our analyzer on sinusoidal currents to validate our extraction method in the general case, when several excitations are emitted with non-unit probability. Sinusoidal drives are well suited to test the robustness of our procedure by comparing our results with parameter-free theoretical predictions. We then apply our technique to trains of Lorentzian pulses carrying an integer charge *q* = −e and *q* = −2e and extract their full content in terms of single-electron wavefunctions. At zero temperature, Lorentzian pulses of integer charge −e*N*_e_ are predicted to generate an integer number *N*_e_ of excitations exclusively above the Fermi sea^6,15–17^. By extracting all the emitted wavefunctions, we observe that thermal effects lead to the generation of a statistical mixture between the expected zero-temperature wavefunctions and additional undesired states. From the measurement of the emission probability of each generated wavefunction, we provide a quantitative analysis of the purity of the generated electronic states. By identifying specific single-electron and hole wavefunctions and determining their emission probabilities for various types of time-dependent currents, our work opens the way to a precise and systematic characterization of quantum information carried by electrical currents.

## Results

### Electronic coherence and Wigner distribution

The main difficulty behind the extraction of the electron and hole wavefunctions from an electrical current lies in the explicit connection between the wavefunctions and a measurable physical quantity. So far, most of the characterizations of the excitations generated by electronic sources have been limited to the measurements of the average electrical current *I*(*t*)^4,18^ and electronic distribution function *f*(*ω*)^7,19^. They provide information on the time and energy distributions, but cannot access the phase of electronic wavefunctions, which requires the use of interferometry techniques.

In analogy with optics, all interference effects are encoded in the first-order electronic coherence ${\cal{G}}_{\rho ,x}^{({\mathrm{e}})}(t,t{\prime})$^20,21^ defined as the time correlations of the fermion field $\hat \Psi (x,t)$, which annihilates an electron at position *x* and time *t* of the one-dimensional conductor: ${\cal{G}}_{\rho ,x}^{({\mathrm{e}})}(t,t{\prime}) = \langle \hat \Psi ^\dagger (x,t{\prime})\hat \Psi (x,t)\rangle _\rho$. To simplify the notations in the rest of the paper, we suppress the superscript (e) and the dependence on the position *x* and on the many-body density operator *ρ* in the expression of the electronic coherence, which is written as ${\cal{G}}(t,t{\prime})$. More generally, ${\cal{G}}(t,t{\prime})$ contains all the information on the single-particle properties of the many-body electronic state. Electronic coherence being a priori a complex function, it is more convenient to use the electronic Wigner distribution^22,23^ *W*(*t*, *ω*) obtained from ${\cal{G}}(t + \tau /2,t - \tau /2)$ by Fourier transform along the time difference *τ*. *W*(*t*, *ω*) is a real function of marginal distributions *I*(*t*) and *f*(*ω*) obtained by respectively integrating *W*(*t*, *ω*) over energy *ω* and time *t*, thereby demonstrating that they only provide partial information.

Subtracting the reference contribution characterized by the zero-temperature Fermi distribution Θ(−*ω*) (where Θ is the Heaviside function) defines Δ_0_*W*(*t*, *ω*) = *W*(*t*, *ω*) − Θ(−*ω*) (or equivalently its Fourier transform ${\mathrm{\Delta }}_0{\cal{G}}(t,t{\prime})$). Δ_0_*W*(*t*, *ω*) and ${\mathrm{\Delta }}_0{\cal{G}}(t,t{\prime})$ are the key quantities that we explicitly connect to the wavefunctions $\varphi _{l,i}^{(\alpha )}$ and emission probabilities $p_i^{(\alpha )}$. This connection is trivial in the pure-state single-body case, that is, when a single excitation (either an electron or a hole) of wavefunction *φ* is emitted with unit probability. In this simple limit, Δ_0_*W*(*t*, *ω*) = *W*_*φ*_(*t*, *ω*), where *W*_*φ*_(*t*, *ω*) is the Wigner representation^24^ of the wavefunction *φ*:

1 $$W_\varphi (t,\omega ) = {\int} {\mathrm{d}} \tau \,\varphi \left( {t + \frac{\tau }{2}} \right)\varphi ^ \ast \left( {t - \frac{\tau }{2}} \right){\mathrm{e}}^{{\mathrm{i}}\omega \tau }.$$

In a recent experiment^25^, Jullien et al. performed the first reconstruction of *W*(*t*, *ω*) in the case of a periodic train of single-electron Lorentzian pulses. Assuming that the single-body limit was valid, they extracted the electronic wavefunction *φ* using Δ_0_*W*(*t*, *ω*) = *W*_*φ*_(*t*, *ω*). However, the single-body limit can never be completely achieved, due to the presence of thermal excitations, to the periodic emission from the source or to deformations of the current pulse associated with imperfections of the voltage drive, or due to more fundamental effects, such as the Coulomb interaction. In addition, for multi-electron states, such as Lorentzian pulses carrying an integer number of excitations *N*_e_ > 1^16,26,27^, going beyond the single-body limit to extract the electronic wavefunctions is absolutely required.

In the more complex case where one has to consider several wavefunctions generated with arbitrary probabilities, only specific sets of drives^28–30^ have been theoretically investigated. Furthermore, the connection between experimentally accessible quantities and the emitted wavefunctions was missing. Following the work of ref. ^14^, we explicitly connect the emitted electron $\varphi _{l,i}^{({\mathrm{e}})}$ and hole $\varphi _{l,i}^{({\mathrm{h}})}$ wavefunctions to the electronic coherence $\Delta _0{\cal{G}}$ by diagonalizing $\Delta _0{\cal{G}}$ in the subspace of electron and hole states (see “Methods”). As a result of the diagonalization procedure, $\Delta _0{\cal{G}}$ can be decomposed on the basis of electron and hole states $\varphi _{l,i}^{(\alpha )}$ by introducing the matrix elements $g_{ij}^{(\alpha \beta )}(l) = \langle \varphi _{l,i}^{(\alpha )}|{\mathrm{\Delta }}_0{\cal{G}}|\varphi _{0,j}^{(\beta )}\rangle = {\int} {\mathrm{d}} t\,{\mathrm{d}}t{\prime}\varphi _{l,i}^{(\alpha )}(t)^ \ast \Delta _0{\cal{G}}(t,t{\prime})\varphi _{0,j}^{(\beta )}(t{\prime})$:

2 $${\Delta _0{\cal{G}}(t,t{\prime})} = \mathop {\sum}\limits_{i = 1}^{N_{\mathrm{e}}} {\mathop {\sum}\limits_{(l,l{\prime}) \in {\Bbb Z}^2} {g_i^{({\mathrm{ee}})}} } (l - l{\prime}){\mkern 1mu} \varphi _{l,i}^{({\mathrm{e}})}(t)\varphi _{l{\prime},i}^{({\mathrm{e}})}(t{\prime})^ \ast \\ - \mathop {\sum}\limits_{i = 1}^{N_{\mathrm{h}}} {\mathop {\sum}\limits_{(l,l{\prime}) \in {\Bbb Z}^2} {g_i^{({\mathrm{hh}})}} } (l - l{\prime}){\mkern 1mu} \varphi _{l,i}^{({\mathrm{h}})}(t)\varphi _{l{\prime},i}^{({\mathrm{h}})}(t{\prime})^ \ast \\ + \mathop {\sum}\limits_{i,j} \mathop {\sum}\limits_{(l,l{\prime}) \in {\Bbb Z}^2} \left( g_{ij}^{({\mathrm{eh}})}(l - l{\prime})\,\varphi _{l,i}^{({\mathrm{e}})}(t)\varphi _{l{\prime},j}^{({\mathrm{h}})}(t{\prime})^ \ast \right. \\ + \left. g_{ji}^{(he)}(l - l{\prime}){\mkern 1mu} \varphi _{l,j}^{({\mathrm{h}})}(t)\varphi _{l{\prime},i}^{({\mathrm{e}})}(t{\prime})^ \ast \right).$$

As the electron and hole wavefunctions $\varphi _i^{(\alpha )}$ are extracted from the diagonalization of $\Delta _0{\cal{G}}$, it naturally implies that there are no quantum coherences in Eq. (2) between states $\varphi _{l,i}^{(\alpha )}$ and $\varphi _{l{\prime},i{\prime}}^{(\alpha )}$ whenever *i* ≠ *i*′: $g_{i \ne i{\prime}}^{(\alpha \alpha )} = 0$.

Each term of Eq. (2) can be separately interpreted. The first (second) term represents the contribution of electron (hole) wavepackets to the first-order coherence. For *l* = *l*′, the real numbers $0 \le g_i^{({\mathrm{ee}})}(0) \le 1$ and $0 \le g_i^{({\mathrm{hh}})}(0) \le 1$ represent the probability for emitting the electron $\left( {\varphi _i^{({\mathrm{e}})}} \right)$ and hole $\left( {\varphi _i^{({\mathrm{h}})}} \right)$ wavefunctions. Following the notation introduced at the beginning of the paper, we thus have $p_i^{(\alpha )} = g_i^{(\alpha \alpha )}(0)$. Compared with the simple picture sketched in Fig. 1, the *T*-periodicity of the source requires to consider also the complex numbers $g_i^{({\mathrm{ee}})}(l - l{\prime})$ (resp. $g_i^{({\mathrm{hh}})}(l - l{\prime})$) for *l* ≠ *l*′ representing coherences between electronic (resp. hole) wavepackets emitted at different periods. The last two terms of Eq. (2) then represent the coherence between the electron and hole states $\varphi _{l,i}^{({\mathrm{e}})}$ and $\varphi _{l{\prime},j}^{({\mathrm{h}})}$ encoded in $g_{ij}^{({\mathrm{eh}})}(l - l{\prime})$. It can only be nonzero when the electron and hole emission probabilities $p_i^{({\mathrm{e}})}$ and $p_j^{({\mathrm{h}})}$ are different from 0 or 1. It then expresses the existence of a quantum superposition between the unperturbed ground state and the creation of the electron/hole pair built from the single-particle states $\varphi _i^{({\mathrm{e}})}$ and $\varphi _j^{({\mathrm{h}})}$. The coefficients $g_{ij}^{({\mathrm{eh}})}(l - l{\prime})$ encode the modulus and phase of such a quantum superposition. In this description, the ideal emission of a quantized number of *N*_e_ electrons and *N*_h_ holes is characterized by $g_i^{({\mathrm{ee}})}(l - l{\prime}) = \delta _{l,l{\prime}}$ and $g_i^{({\mathrm{hh}})}(l - l{\prime}) = \delta _{l,l{\prime}}$ implying that $g_{ij}^{({\mathrm{eh}})}(l - l{\prime}) = 0$.

This formalism serves as the theoretical background for the extraction of the electron and hole wavefunctions from experimental measurements. Using two-particle interferences, we proceed to the measurement of the electronic coherence Δ_0_*W* and $\Delta _0{\cal{G}}$ for arbitrary electrical currents. We then implement an algorithm (see "Methods"), which identifies the emitted wavefunctions $\varphi _i^{({\mathrm{e}})}$ and $\varphi _j^{({\mathrm{h}})}$ from the diagonalization of $\Delta _0{\cal{G}}$ in the subspace of electron and hole states and recasts it in the form given by Eq. (2). This set of data describes completely the single-particle content of the electronic current and quantifies how far it deviates from the ideal emission regime.

### Experimental setup and protocol

**Fig. 2:**
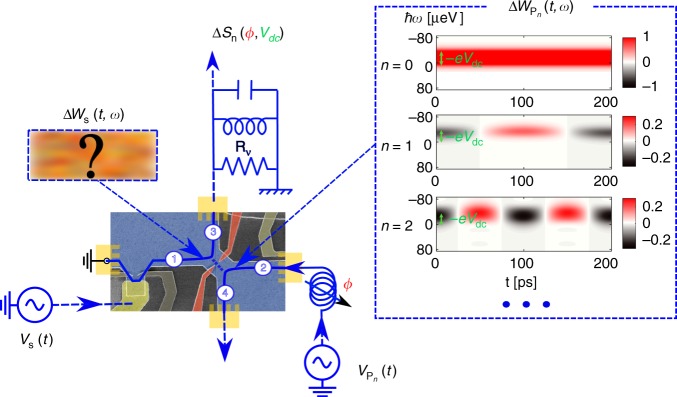
Sketch of the experimental setup. The two-dimensional electron gas is represented in blue color and the edge channels as blue lines. A quantum point contact (red color) is used as an electronic beam splitter. A time-dependent voltage *V*_S_(*t*) is applied to a mesoscopic capacitor (gate in gold color capacitively coupled to the edge channel) placed at input 1 of the beam splitter and generates the unknown Wigner distribution Δ*W*_S_(*t*, *ω*). The probe signal $V_{{\mathrm{P}}_n}(t)$, a low-amplitude sinusoidal drive at frequency *nf* is generated at input 2. The corresponding probe Wigner distributions $\Delta W_{{\mathrm{P}}_n}(t,\omega )$ are plotted for *n*  =  0 to *n *= 2 (the frequency is *f* = 5 GHz and the temperature *T*_el_ = 80 mK). The current noise at the output of the splitter is converted to a voltage noise on the quantized resistance *R*_*ν *_*=* *h*/(*ν*e^2^). *R*_*ν*_ is connected to an LC tank circuit used to shift the measurement frequency at the resonance *f*_0 _= 1.45 MHz

The experiment is performed in a high-mobility GaAs/AlGaAs two-dimensional electron gas placed in a strong perpendicular magnetic field so as to reach the quantum Hall regime at filling factors *ν* = 2 or *ν* = 3, where charge propagates along one-dimensional chiral-edge channels. We focus on the propagation on the outer-edge channel, which realizes a ballistic spin-polarized one-dimensional conductor. The electronic source is a metallic gate capacitively coupled to the edge channels, allowing us to shape any charge distribution^31^ by applying the proper time-dependent voltage to the gate. The resulting Wigner distribution *W*_S_(*t*, *ω*) can be reconstructed^32^ by measuring two-electron interferences^33,34^, using an electronic Hong–Ou–Mandel^35^ interferometer^36–38^. As shown in Fig. 2, the interferometer consists of a quantum point contact used as an electronic beam splitter partitioning the excitations propagating from inputs 1 and 2 with transmission probability ${\cal{T}}$. Input 1 is connected to the source, whereas input 2 is connected to a voltage-driven ohmic contact that will generate a set of known reference states, called probe states, of Wigner distribution $W_{{\mathrm{P}}_n}$ for $n \in {\Bbb N}$. For each probe state, we measure the excess noise Δ*S*_*n*_ at output 3 between the source being switched on and off^13^:

3 $$\Delta S_n = \,\, 2{\mathrm{e}}^2{\cal{T}}(1 - {\cal{T}}){\int} {\frac{{d\omega }}{{2\pi }}} \left[ {\overline {\Delta W_{\mathrm{S}}(t,\omega )} ^t(1 - 2f_{{\mathrm{eq}}}(\omega ))} \right. \\ \,\,\,\,- \left. {2\overline {\Delta W_{\mathrm{S}}(t,\omega )\Delta W_{{\mathrm{P}}_n}(t,\omega )} ^t} \right]$$

where $\overline \cdots ^t$ denotes the average over time *t*, and $\Delta W_{{\mathrm{S}}/{\mathrm{P}}_n}$ are, respectively, the source and probe excess Wigner distributions with respect to the Fermi–Dirac distribution *f*_eq_(*ω*) at temperature *T*_el_ ≠ 0: $W_{{\mathrm{S}}/{\mathrm{P}}_n}(t,\omega ) = f_{{\mathrm{eq}}}(\omega ) + {\mathrm{\Delta }}W_{{\mathrm{S}}/{\mathrm{P}}_n}(t,\omega )$. The first term in Eq. (3) represents the classical random partition noise of the source. It is reduced by the second term in Eq. (3), which represents the antibunching between indistinguishable source and probe excitations colliding on the splitter. Their degree of indistinguishability is given by the overlap between Δ*W*_S_ and $\Delta W_{{\mathrm{P}}_n}$. By properly choosing the set of probe states, Eq. (3) allows for the reconstruction of any unknown Wigner distribution^22,32^.

A convenient set of probe states can be used to reconstruct each harmonic of the Fourier expansion of the excess source Wigner distribution

4 $$\Delta W_{\mathrm{S}}(t,\omega ) = \mathop {\sum}\limits_{n \in {\Bbb Z}} \Delta W_{{\mathrm{S}},n}(\omega )\,{\mathrm{e}}^{2\pi {\mathrm{i}}nft},$$

where *f* = 1/*T* denotes the driving frequency. The *n* = 0 harmonic represents the source excess electronic distribution function Δ*f*(*ω*). All the time dependence of Δ*W*_S_(*t*, *ω*) is encoded in the *n* ≠ 0 harmonics. To select the contribution from the *n*th harmonic in Eq. (3), we apply on the probe input a small ac signal at frequency *nf* on top of a dc bias^32^: $V_{{\mathrm{P}}_n}(t) = V_{{\mathrm{dc}}} + V_{{\mathrm{P}}_n}cos(2\pi nft + \varphi )$. The resulting Wigner distribution $W_{{\mathrm{P}}_n}$ (plotted in Fig. 2) evolves periodically in time at frequency *nf*. By measuring the output noise Δ*S*_*n*_ as a function of *ϕ* and *V*_dc_ (see “Methods”), the real and imaginary parts of Δ*W*_S*,n*_(*ω*) can be extracted.

### Electronic Wigner distribution of sinusoidal drives

**Fig. 3:**
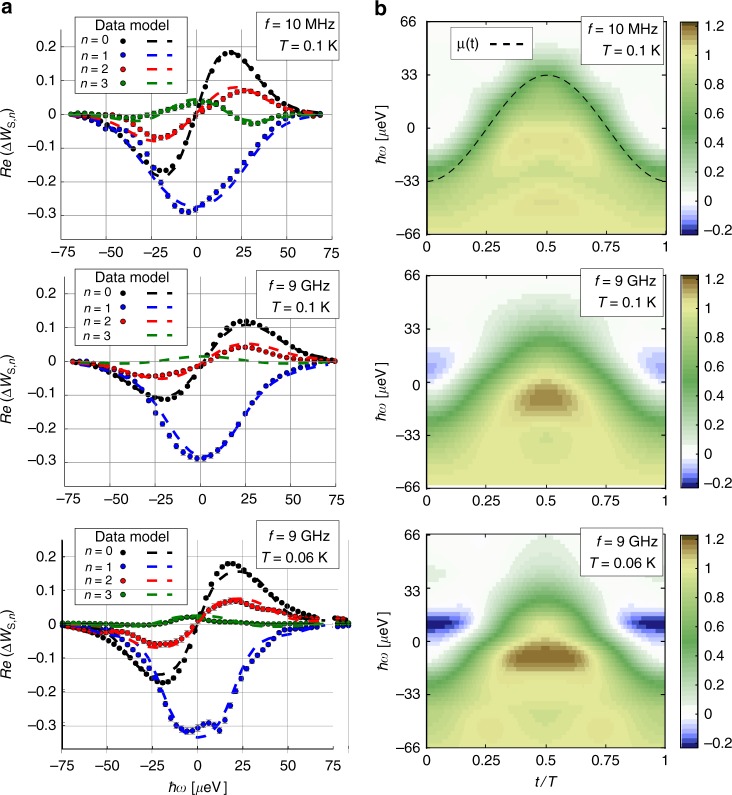
Wigner distribution of sinusoidal drives. **a** Measured Δ*W*_S,*n*_(*ω*) for *n *= 0 to *n *= 3 for sinusoidal drives at frequency *f*  = 10 MHz and *f* = 9 GHz. The observed parity: Δ*W*_S,*n*_(−*ω*) = (−1)^*n*+1^Δ*W*_S,*n*_(*ω*), directly stems from the electron/hole symmetry of the sinusoidal drive. Error bars are defined as standard error of the mean. **b** Time–energy representation of the Wigner distribution *W*_S_(*t*, *ω*)

We first apply our quantum current analyzer to sinusoidal drives, *V*_S_(*t*) = *V*_S_ cos(2*πft*) at various frequencies *f*. Figure 3a presents the measurements of the *n* = 0, 1, 2, 3 harmonics of $\Re (\Delta W_{{\mathrm{S}},n})$ (ℑ(Δ*W*_S,*n*_) = 0) of three sinusoidal drives of similar amplitudes (*V*_S_≈32 μV). We first focus on the effect of frequency by comparing Δ*W*_S,*n*_ for *f* = 10 MHz and *f* = 9 GHz at *T*_el_ = 100 mK. The *n* = 0, 2, and 3 harmonics are lower for *f* = 9 GHz compared with *f* = 10 MHz (Δ*W*_S,*n*=3_ even falls below our experimental resolution for *f*  = 9 GHz).

Indeed, in a photo-assisted description of electronic transport^39,40^, the *n* ≠ 1 harmonics are related to multiphoton absorption/emission processes, whose strength increases with the ratio *eV*_S_/*hf*, which equals 800 for *f* = 10 MHz compared with 0.8 for *f* = 9 GHz. We then turn to the effect of temperature by comparing Δ*W*_S,*n*_ for the two drives at *f* = 9 GHz, but at different temperatures. Decreasing the temperature from *T*_el_ = 100 mK to *T*_el_ = 60 mK leads to a narrowing of all the harmonics and to an increase of their amplitude. For the three drives, the agreement between the data and theoretical predictions (dashed lines) is excellent, showing the robustness of our reconstruction procedure. After measuring all relevant Δ*W*_S,*n*_, we can combine them in Eq. (4) to reconstruct *W*_S_(*t*, *ω*).

The Wigner distributions are represented in Fig. 3b. Within experimental accuracy, the *f* = 10-MHz case follows an equilibrium distribution function, *W*_S_(*t*, *ω*) = *f*_eq,*μ*(*t*)_(*ω*), with a time-varying chemical potential following the ac drive: *μ*(*t*) = −eV_S_ cos (2*πft*). This is expected as the *f* = 10 MHz case corresponds to a quasi-classical current (*hf* ≪ *k*_B_*T*_el_) characterized by bounded values of the Wigner distribution, 0 ≤ *W*(*t*, *ω*) ≤ 1, such that *W*(*t*, *ω*) can be interpreted as a time-dependent electronic distribution function and viewed as an adiabatic evolution of the stationary (dc) case^22^. In contrast, *hf*≥*k*_B_*T*_el_ corresponds to the quantum case, where the Wigner distribution can take negative or above one values. This is what we observe for the *f* = 9-GHz drives, with a strong emphasis of these quantum features at the lowest temperature *T*_el_ = 60 mK. Consequently, in the quantum regime, single-particle properties are no longer described in terms of a time-varying electronic distribution function. This is the case where *W*(*t*, *ω*) can be used to extract electron and hole wavefunctions.

### Electron/hole wavefunctions generated by sinusoidal drives

**Fig. 4:**
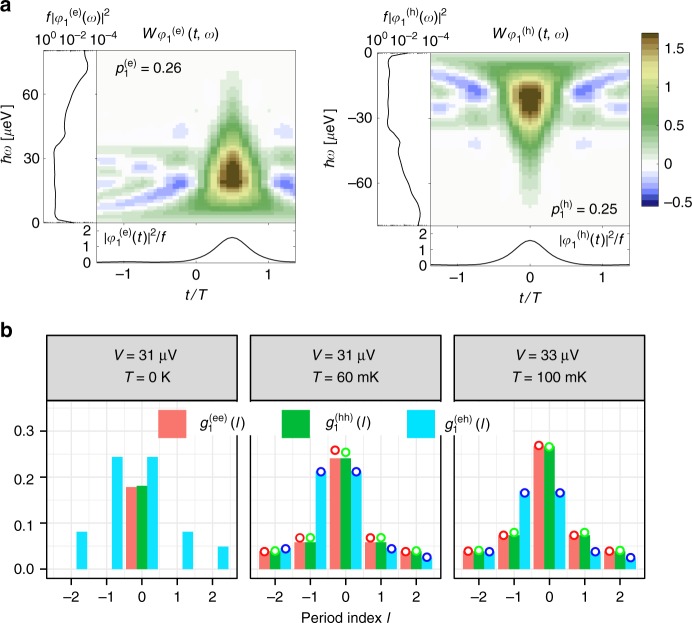
Electron and hole wavefunctions generated by low-amplitude sinusoidal drives. **a** Wigner distribution functions $W_{\varphi _1^{({\mathrm{e/h}})}}(t,\omega ) = {\int} d \tau \varphi _1^{({\mathrm{e/h}})}\left( {t + \frac{\tau }{2}} \right)\varphi _1^{({\mathrm{e/h}}) \ast }\left( {t - \frac{\tau }{2}} \right){\kern 1pt} {\mathrm{e}}^{{\mathrm{i}}\omega \tau }$ for the dominant electronic $\varphi _1^{({\mathrm{e}})}$ and hole $\varphi _1^{({\mathrm{h}})}$ wavefunctions for *f* = 9 GHz and *T*_el _= 60 mK ($\varphi _1^{({\mathrm{e/h}})}$ obtained at *T*_el_ = 100 mK are almost identical). The panels in the margins of the color plots represent the time $|\varphi _1^{({\mathrm{e/h}})}(t)|^2/f$ and energy $f|\varphi _1^{({\mathrm{e/h}})}(\omega )|^2$ distributions obtained by integrating $W_{\phi _1^{({\mathrm{e/h}})}}(t,\omega )$ over *ω* and *t*. **b** Moduli of the interperiod coherence |*g*^(ee)^(*l*)|, |*g*^(hh)^(*l*)| and |*g*^(eh)^(*l*)| (colored bars correspond to numerical calculations and colored dots to the experimental data)

The second step of our analyzer extracts individual electronic wavepackets from the reconstructed Wigner distribution by implementing an algorithm (see "Methods"), which recasts our measurements in the form of Eq. (2). Figure 4 presents the result of this analysis on the experimental data obtained for the *f* = 9-GHz sinusoidal drives. As the probability to emit more than one electron/hole is very small, the analysis can be limited to one electron $\varphi _1^{({\mathrm{e}})}$ and one hole $\varphi _1^{({\mathrm{h}})}$ wavefunction $\left( {p_{i > 1}^{(\alpha )} \approx 10^{ - 3} \ll 1} \right)$. They are plotted in the Wigner representation in the case *T*_el_ = 60 mK in Fig. 4a. The hole is shifted by half a period with respect to the electron and its energy distribution $|\varphi _1^{({\mathrm{h}})}(\omega )|^2$ mirrors that of the electron’s at positive energy. As a figure of merit of the procedure, we evaluate the state fidelity defined as the overlap between electron and hole wavefunctions extracted from the experimental data and the electron and hole wavefunctions extracted from numerical computations of the Wigner distribution using Floquet scattering theory (see Supplementary Note 1). The results are in excellent agreement with a fidelity >0.99 for all the extracted wavefunctions, demonstrating the accuracy of the state reconstruction.

Figure 4b depicts the moduli of the inter-period coherences between emitted wavepackets for different temperatures (bars represent numerical simulations, circles represent data). When the temperature increases, the occupation probabilities which, in the present case, are very close due to electron/hole symmetry $\left( {p_1^{({\mathrm{e}})} \approx p_1^{({\mathrm{h}})}} \right)$ increase from 0.17 (numerical calculation at *T*_el_ = 0 K) to 0.25 (60 mK) and 0.27 (100 mK). Coherences between different periods ($g_1^{({\mathrm{ee}})}(l \ne 0)$ and $g_1^{({\mathrm{hh}})}(l \ne 0)$) also appear and extend over the thermal coherence time ($h/k_{\mathrm{B}}T_{{\mathrm{el}}} \simeq 0.5\,{\mathrm{ns}}$ at 100 mK). This reflects that at finite temperature, the electron and hole states $\varphi _1^{({\mathrm{e/h}})}$ have a finite probability to be occupied by thermal excitations. As the probability to emit the electron and hole differ from 1, we also observe nonzero electron/hole coherence: $g_{11}^{({\mathrm{eh}})}(l - l{\prime}) \ne 0$. Interestingly, these terms are suppressed by thermal fluctuations, reflecting the transition from a pure quantum state at *T*_el_ = 0 K to a statistical mixture at higher temperature. At *T*_el_ = 0 K, a single process occurs: the generation of the quantum superposition between the unperturbed ground state (with probability $1 - p_1^{({\mathrm{e}})}$) and the creation of the electron/hole pair (with probability $p_1^{({\mathrm{e}})}$). Thermal fluctuations allow two additional processes: only the electron state, or only the hole state, can be generated. The resulting state at finite temperature is a statistical mixture between these three processes. Our algorithm enables a quantitative description by computing a purity indicator, ${\Bbb P}$, from the extracted inter-period coherences (see “Methods”). It quantifies the weight of coherent electron/hole processes with respect to all emitted excitations. By construction, ${\Bbb P} = 1$ at zero temperature, and from our experimental data, decreases to 0.71 at *T*_el_ = 60 mK and to 0.58 at *T*_el_ = 100 mK. Numerical evaluation of the same quantity calculated using Floquet scattering theory (see Supplementary Note 1) give 0.999 at zero temperature, 0.725 at 60 mK and 0.588 at 100 mK in very good agreement with the experimental data.

### Single-electron Lorentzian pulse (*q* = −e)

We now turn to the analysis of a current generated by periodic Lorentzian voltage pulses $V_{\mathrm{S}}(t) = \mathop {\sum}\nolimits_l - \frac{{V_0}}{{1 + (t - lT)^2/\tau ^2}}$ with *τ* = 42 ps, *f* = 4 GHz, and *V*_0_ chosen such that each pulse carries exactly a single-electron charge: $\frac{{{\mathrm{e}}^2}}{h}{\int}_0^T {V_{\mathrm{S}}} (u)du = - {\mathrm{e}}$. The Lorentzian pulses are generated by calibrating the amplitude and phase of each harmonic of the current at the location of the beam splitter (see “Methods”). This ensures that phase and amplitude shifts caused by Coulomb interaction effects^26,41^ during the propagation along the edge channels can be absorbed in the calibration process. This procedure allows us to demonstrate the proof of principle of our quantum current tomography without having to consider Coulomb interaction effects.

**Fig. 5:**
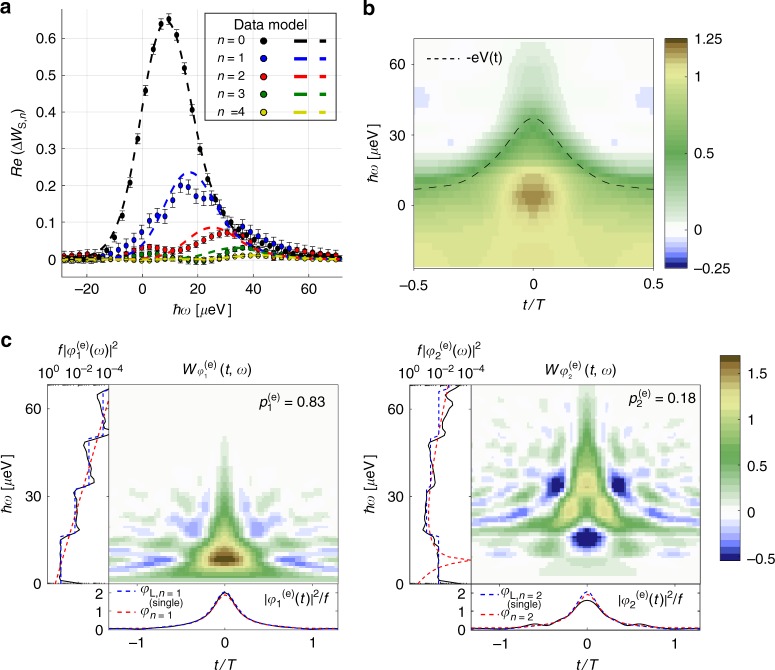
Electron wavefunction generated by a Lorentzian pulse, *q = *−e. **a** Measured Δ*W*_S,*n*_(*ω*) (*n *= 0 to *n *= 4) for the single-electron Lorentzian pulse at *f *= 4 GHz, *τ *= 42 ps, and *T*_el _= 50 mK. Error bars are defined as standard error of the mean. **b** *W*_S_(*t*, *ω*), the dashed line represents the voltage pulse *V*(*t*) = *h*/e^2^*I*(*t*), where *I*(*t*) is obtained by integrating *W*_S_(*t*, *ω*) on energy *ω*. **c** Wigner representation of $\varphi _1^{({\mathrm{e}})}(t)$ (left) and $\varphi _2^{({\mathrm{e}})}(t)$ (right). The panels in the margins represent the time $|\varphi _i^{({\mathrm{e}})}(t)|^2$ and energy $|\varphi _i^{({\mathrm{e}})}(\omega )|^2$ distributions obtained by integrating $W_{\varphi _i^{({\mathrm{e}})}}(t,\omega )$ over *ω* and *t*. $|\varphi _1^{({\mathrm{e}})}(\omega )|^2$ is represented in log scale for better comparison with theoretical predictions with single shot $\varphi _n^{({\mathrm{single}})}$ (red dashed line) and periodic *φ*_L,*n*_ (blue dashed line) Lorentzian wavepackets (with *τ = *42 ps). As it can be seen from the very good agreement with the blue dashed line, the steplike behavior of $|\varphi _1^{({\mathrm{e}})}(\omega )|^2$ comes from the periodicity of the drive

The measured *n* = 0 to *n* = 4 harmonics of Δ*W*_S_(*t*, *ω*) are plotted in Fig. 5a. They are located on the positive energy side with maxima shifted by *nhf*/2e for increasing *n* and, indeed, take very small value for |*ω*| ≤ *nπf* showing that almost no hole excitation is emitted. Their energy width is imposed by the temporal width of the pulse *τ* and their amplitude reproduces the decrease of the harmonics of *I*(*t*) (obtained by integration of Δ*W*_S,*n*_(*ω*)). The overall agreement with theoretical predictions (dashed lines) is good (no fitting parameter). The resulting Wigner distribution *W*_*S*_(*t*, *ω*) is plotted in Fig. 5b. Strong nonclassical features (*W*_S_(*t*, *ω*) ≈ 1.2) are observed at the location of the electron excitation in the (*t*, *ω*) plane.

The wavefunctions extracted from our analysis are plotted in Fig. 5c. First, as expected for a single-electron Lorentzian pulse, this analysis quantitatively confirms that almost no hole excitation is emitted: *p*^(h)^ = 0.03 ± 0.01. Second, contrary to the previous case (low-amplitude sine drive), two electronic wavefunctions $\varphi _1^{({\mathrm{e}})}$ and $\varphi _2^{({\mathrm{e}})}$ contribute with emission probabilities $p_1^{({\mathrm{e}})} = 0.83 \pm 0.01$ and $p_2^{({\mathrm{e}})} = 0.18 \pm 0.01$. This means that finite temperature (depending on the ratio *k*_B_*T*_el_/*hf*, see Supplementary Note 2) leads to the generation of a statistical mixture^42^ (of purity ${\Bbb P} = 0.75$) between $\varphi _1^{({\mathrm{e}})}$ and $\varphi _2^{({\mathrm{e}})}$ with weights $p_1^{({\mathrm{e}})}$ and $p_2^{({\mathrm{e}})}$ $\left( {p_1^{({\mathrm{e}})} + p_2^{({\mathrm{e}})} = 1.01 \pm 0.01} \right)$. Note that such an emission of a statistical mixture between different single-electron wavefunctions could not be captured within the single-body limit considered in the previous analysis of Jullien et al.^25^, which underlines the need for developing the general approach we demonstrate here.

The wavefunctions $\varphi _1^{({\mathrm{e}})}$ and $\varphi _2^{({\mathrm{e}})}$ can be compared with the expected ones generated by Lorentzian voltage pulses at zero temperature. A single Lorentzian pulse of temporal width *τ* and carrying an integer number *N*_e_ of electrons is the Slater determinant built over the 1 ≤ *n* ≤ *N*_e_ electronic wavefunctions^16,26^ $\varphi _n^{({\mathrm{single}})}(\omega ) = \frac{1}{{\sqrt {\cal{N}} }}\Theta (\omega )\exp ( - \omega \tau ){\cal{L}}_{n - 1}(2\omega \tau )$, where ${\cal{N}}$ is a normalization constant, ${\cal{L}}_n$ is the *n*th Laguerre polynomial. The two first ones $\varphi _{n = 1}^{({\mathrm{single}})}(\omega )$ and $\varphi _{n = 2}^{({\mathrm{single}})}(\omega )$, plotted in red dashed lines in Fig. 5c, are very similar to $\varphi _1^{({\mathrm{e}})}$ and $\varphi _2^{({\mathrm{e}})}$. However, they do not reproduce the discretization of the energy distribution in units of *hf*. This discretization is related to the periodicity of the single-electron emission, which is not captured by the expression of $\varphi _n^{({\mathrm{single}})}$. The *n* = 1 wavefunction of the periodic train of Lorentzian pulses has been shown^14,43^ to be given by $\varphi _{{\mathrm{L}},n = 1}(\omega ) = \frac{1}{{\sqrt {\cal{N}} }}\Theta (\omega )\exp ( - \omega _1\tau )$ where $\omega _1 = 2\pi f\left\lfloor {\frac{\omega }{{2\pi f}}} \right\rfloor$ has quantized steps 2*πf* related to the pulse periodicity. Here, we generalize this expression to the *n* = 2 and *n* = 3 wavefunctions by using the following ansatz: $\varphi _{{\mathrm{L}},n}(\omega ) = \frac{1}{{\sqrt {\cal{N}} }}\Theta (\omega )\exp ( - \omega _n\tau ){\cal{L}}_{n - 1}(2\omega _n\tau )$ where $\omega _n = 2\pi f\left( {x_n + \left\lfloor {\frac{\omega }{{2\pi f}}} \right\rfloor } \right)$. We take *x*_1_ = 0 following refs. ^14,43^ and then numerically deduce *x*_2_ = 0.33 and *x*_3_ = 0.24 from the constraint that the wavefunctions should be orthogonal: 〈*φ*_L,*n*_|*φ*_L,*n*′_〉 = *δ*_*n*,*n*′_. Comparing the wavefunctions $\varphi _1^{({\mathrm{e}})}$ and $\varphi _2^{({\mathrm{e}})}$ extracted from our experimental data to these theoretical predictions, we observe that $\varphi _1^{({\mathrm{e}})}$ is very close to the expected wavefunction *φ*_L,*n*=1_ (blue dashed line in Fig. 5c) with an overlap of 0.98. Interestingly, $\varphi _2^{({\mathrm{e}})}$ which is emitted at higher energy strongly resembles *φ*_L,*n*=2_ with an overlap of 0.93 leading to this simple interpretation of temperature effects: finite temperature leads to the emission of a statistical mixture between the expected *n* =1 Lorentzian wavefunction and the wavefunctions corresponding to higher excitations numbers *n* > 1. Importantly, our observations are not related to imperfections of the emission drive or to errors of our extraction method, but only to thermal effects. This probabilistic description of the electron state stems from the nonzero entropy of the finite-temperature ground state, which reveals the statistical (non-quantum) fluctuations of the ground state. This interpretation is confirmed by numerical calculations. Applying our method on perfect periodic Lorentzian pulses calculated using Floquet scattering theory (see Supplementary Note 2), we recover that for *T*_el_ = 0 K, $p_1^{({\mathrm{e}})} = 1$ and $p_2^{({\mathrm{e}})} = 0$ (pure state) but for *T*_el_ = 50 mK, $p_1^{({\mathrm{e}})} = 0.84$ and $p_2^{({\mathrm{e}})} = 0.18$, in very good agreement with our experimental results.

### Two-electron Lorentzian pulse (*q* = −2e)

**Fig. 6:**
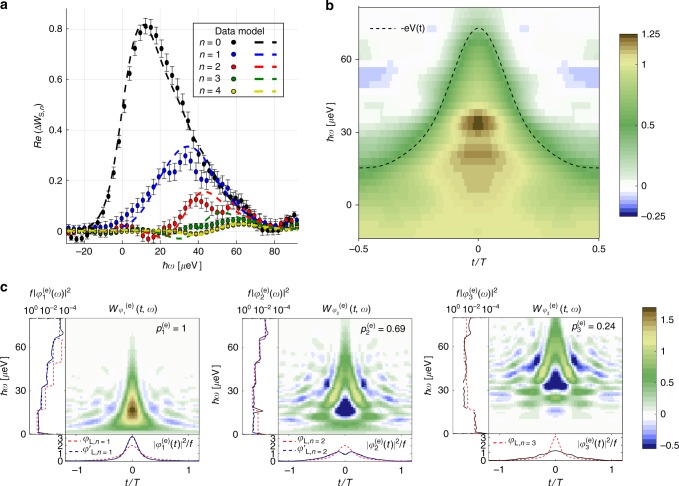
Electron wavefunction generated by a Lorentzian pulse, *q *= −2e. **a** Measured Δ*W*_S,*n*_(*ω*) (*n *= 0 to *n* = 4) for the two-electron Lorentzian pulse at *f* = 4 GHz, *τ *= 42 ps, and *T*_el_ = 50 mK. Error bars are defined as standard error of the mean. **b** *W*_S_(*t*, *ω*), the dashed line represents the voltage pulse *V*(*t*) = *h*/e^2^*I*(*t*), where *I*(*t*) is obtained by integrating *W*_S_(*t*, *ω*) on energy *ω*. **c** Wigner representation of $\varphi _1^{({\mathrm{e}})}(t)$ (left) and $\varphi _2^{({\mathrm{e}})}(t)$ (middle) and $\varphi _3^{({\mathrm{e}})}(t)$ (right). The panels in the margins represent the time $|\varphi _i^{({\mathrm{e}})}(t)|^2$ and energy $|\varphi _i^{({\mathrm{e}})}(\omega )|^2$ distributions obtained by integration of $W_{\varphi _i^{({\mathrm{e}})}}(t,\omega )$ over *ω* and *t*. $|\varphi _1^{({\mathrm{e}})}(\omega )|^2$ is represented in log scale. The red dashed line represent the theoretical predictions for the *n *= 1 to *n *= 3 wavefunctions of periodic trains of Lorentzian pulses *φ*_L,*n*_. The blue dashed lines represent the theoretical predictions for *φ*′_L,*n*_ obtained from linear combinations of the *φ*_L,*n*_

Finally, we analyze periodic trains of Lorentzian pulses carrying the charge of two electronic excitations: $V_{\mathrm{S}}(t) = \mathop {\sum}\limits_l - \frac{{2V_0}}{{1 + (t - lT)^2/\tau ^2}}.$ Our experimental results are plotted in Fig. 6. As in the single-electron case, thermal effects lead to the generation of a statistical mixture of different wavefunctions, one more than the number of emitted charges. The first wavefunction $\varphi _1^{({\mathrm{e}})}$ is emitted with unit probability $p_1^{({\mathrm{e}})} = 1,$ but $\varphi _2^{({\mathrm{e}})}$ and $\varphi _3^{({\mathrm{e}})}$ are emitted with probabilities smaller than one, $p_2^{({\mathrm{e}})} = 0.69 \pm 0.02$ and $p_3^{({\mathrm{e}})} = 0.24 \pm 0.02$, reflecting that the emitted state is a statistical mixture of purity ${\Bbb P} = 0.68$.

Interestingly, and contrary to the *q* = −e, case, $\varphi _1^{({\mathrm{e}})}$ and $\varphi _2^{({\mathrm{e}})}$ do not correspond to the expected Lorentzian wavefunctions *φ*_L,*n*=1_ and *φ*_L,*n*=2_ plotted in red dashed line in Fig. 6c. This can be understood by discussing first the zero-temperature case. At *T*_el_ = 0 K, the generated state is predicted to be described by the Slater determinant formed from the wavefunctions *φ*_L,*n*=1_ and *φ*_L,*n*=2_. However, any choice of basis obtained by a linear combination of *φ*_L,*n*=1_ and *φ*_L,*n*=2_ is equally valid to describe this Slater determinant (see Supplementary Note 3). At finite temperature, *T*_el_ ≠ 0 K, this ambiguity in the choice of the two wavefunctions describing the electronic state is lifted as $\varphi _1^{({\mathrm{e}})}$ and $\varphi _2^{({\mathrm{e}})}$ are no longer generated with the same probability: $p_1^{({\mathrm{e}})} \ne p_2^{({\mathrm{e}})}( \ne 1)$.

Searching for the basis of states which maximizes the overlap with $\varphi _1^{({\mathrm{e}})}$ and $\varphi _2^{({\mathrm{e}})}$, we observe that finite temperature favors the emergence of two specific states obtained by the following linear combination of *φ*_L,*n*=1_ and *φ*_L,*n*=2_:

5 $$\varphi{\prime}_{{\mathrm{L}},n = 1} = \cos \left( {\frac{\theta }{2}} \right){\mkern 1mu} \varphi _{{\mathrm{L}},n = 1} + \sin \left( {\frac{\theta }{2}} \right){\mkern 1mu} \varphi _{{\mathrm{L}},n = 2}$$

6 $$\varphi{\prime}_{{\mathrm{L}},n = 2} = \sin \left( {\frac{\theta }{2}} \right)\;\varphi _{{\mathrm{L}},n = 1} - \cos \left( {\frac{\theta }{2}} \right){\mkern 1mu} \varphi _{{\mathrm{L}},n = 2}$$

with $\theta \sim \frac{\pi }{2} \times 0.37$. The single-electron wavefunctions $\varphi{\prime}_{{\mathrm{L}},n = 1}$ and $\varphi{\prime}_{{\mathrm{L}},n = 2}$, plotted in blue dashed lines in Fig. 6c, have a very strong overlap with $\varphi _1^{({\mathrm{e}})}$ and $\varphi _2^{({\mathrm{e}})}$ (0.99 and 0.96). As $p_1^{({\mathrm{e}})} = 1$ and $p_2^{({\mathrm{e}})} = 0.69$, it means that with probability 0.69 the two electron state described by the Slater determinant formed from $\varphi{\prime}_{{\mathrm{L}},1}$ and $\varphi{\prime}_{{\mathrm{L}},2}$ is generated. This state is equivalent to the expected Slater determinant formed from *φ*_L,1_ and *φ*_L,2_. However, with probability $p_2^{({\mathrm{e}})} = 0.24$ a different two electron state is generated corresponding to the Slater determinant formed from $\varphi{\prime}_{{\mathrm{L}},1}$ and $\varphi _3^{({\mathrm{e}})}$.

The connection between $\varphi _3^{({\mathrm{e}})}$ and a theoretically predicted wavefunction is less clear. $|\varphi _3^{({\mathrm{e}})}(\omega )|^2$ resembles |*φ*_L,*n*=3_(*ω*)|^2^, the energy distribution of the *n* = 3 Lorentzian wavefunction (blue dashed lines in Fig. 6c). However the time distributions are different, which is reflected by the relatively small overlap of 0.86 between the two states.

As for the *q* = −e case, we can check using numerical computations that the generation of a statistical mixture between two different Slater determinants is caused by the finite temperature. The simulations presented in Supplementary Note 3 confirm that the probability to generate the Slater determinant formed from *φ*_L,1_ and *φ*_L,2_ decreases from 1 at zero temperature to 0.79 at *T*_el_ = 50 mK, while the probability to generate the Slater determinant formed from $\varphi{\prime}_{{\mathrm{L}},1}$ and $\varphi _3^{({\mathrm{e}})}$ increases from 0 at zero temperature to 0.18 at *T*_el_ = 50 mK in reasonable agreement with our observations.

## Discussion

We have demonstrated a quantum tomography protocol for arbitrary electrical currents. Without any a priori knowledge on the electronic state, this protocol extracts all the electron and hole wavefunctions and their emission probabilities.

Our protocol is the tool of choice for characterizing single- to few-electron sources by extracting all the *N*_e_ single-electron wavefunctions generated at each period. We have analyzed in this work the *N*_e_ = 1 and *N*_e_ = 2 Lorentzian voltage pulses and have extracted the wavefunctions of each generated electronic excitation. The generation of Lorentzian electronic wavepacket in a ballistic one-dimensional channel, which we demonstrate here, is an important milestone for the development of time-resolved quantum electronics. Numerous recent proposals suggest the use of time-resolved charge or energy currents^44^ carried by integer charge Lorentzian voltage pulses to probe the timescales of quantum coherent conductors^45^ or to dynamically control their interference pattern^46^.

The reconstruction of the quantum state of single electronic excitations is a currently active research field as illustrated by the very recent achievement of the state tomography of high energy electrons propagating along quantum Hall edge channels^47,48^. These recent works highlight the importance of characterizing the purity of the emitted states. Importantly, our protocol fully captures the differences between pure states and statistical mixtures and provides a quantitative evaluation of the purity. This ability to quantify the purity of quantum states generated by electronic sources is crucial for future applications of quantum electronics. More specifically, we find that for single-charge Lorentzian pulses, which are predicted to generate a pure single-electron wavefunction at zero temperature, poisoning by thermal excitations results in the emission of a mixture (with purity ${\Bbb P} = 0.75$) of two different states, which correspond to the *n* = 1 and *n* = 2 Lorentzian wavepackets *φ*_L,*n*_. For two-electron Lorentzian pulses, we show that thermal effects lead to the generation of a mixture between the zero temperature Slater determinant formed from *φ*_L,1_ and *φ*_L,2_ and an undesired two electron state which we fully characterize.

The generation and characterization of multi-electron states in quantum conductors also opens the way to the study of correlations and interactions between a controlled number of excitations emitted on demand in the circuit, with applications to the controlled generation of entangled electron or electron/hole^49^ pairs. In this context, this protocol can also be applied to identify single-particle wavefunctions generated in interacting conductors^41^ and supplemented by other measurements^50^, to quantify the importance of interaction-induced quantum correlations.

Finally, it can establish a bridge between electron and microwave quantum optics^51,52^ by probing the electronic content of microwave photons injected from a transmission line into a quantum conductor.

## Methods

### Sample and noise measurements

The sample is a GaAs/AlGaAs two-dimensional electron gas of charge density *n*_s_ = 1.9 × 10^15^ m^−2^ and mobility *μ* = 2.4 × 10^6^ cm^−2^ V^−1^ s^−1^. It is placed in a high magnetic field to reach the quantum Hall regime at filling factor *ν* = 2 (*B* = 3.7 T) and *ν* = 3 (*B* = 2.6 T). The measurements of the Wigner distribution of sinusoidal drives have been performed at *ν* = 2. The measurements of the Wigner distributions of Lorentzian pulses have been performed at *ν* = 3. The current noise at the output of the quantum point contact is converted to a voltage noise via the quantum Hall edge channel resistance *R*_*ν*_ = *h*/*ν*e^2^ between output 3 and the ohmic ground (see Fig. 2). In order to move the noise measurement frequency in the MHz range, *R*_*ν*_ is connected to an LC tank circuit of resonance frequency *f*_0_ = 1.45 MHz. The tank circuit is followed by a pair of homemade cryogenic amplifiers followed by room-temperature amplifiers. A vector signal analyzer measures the correlations between the voltages at the output of the two amplification chains in a 78-kHz bandwidth centered on *f*_0_. The noise measurements are calibrated by measuring both the thermal noise of the output resistance *R*_*ν*_, and the partition noise of a d.c. bias applied on input 2 of the electronic beam splitter.

### Generation of Lorentzian current pulses

The single-electron and two-electron periodic trains of Lorentzian current pulses are generated by applying the ac part of the signal *V*_ac_(*t*) on the mesoscopic capacitor placed at input 1 of the beam splitter, see Fig. 1, and the dc part of the signal *V*_dc_ on the ohmic contact connected to input 1 of the beam splitter such that $V_{\mathrm{S}}(t) = V_{{\mathrm{ac}}}(t) + V_{{\mathrm{dc}}} = \mathop {\sum}\limits_l - \frac{{V_0}}{{1 + (t - lT)^2/\tau ^2}}$. More precisely, *V*_ac_(*t*) is generated harmonic by harmonic (from *n* = 1 to *n* = 5): $V_{{\mathrm{ac}}}(t) = \mathop {\sum}\limits_{n = 1}^{n = 5} {V_{{\mathrm{ac}},n}} \cos (n\Omega t + \phi _n)$. The careful calibration of the amplitude *V*_ac,*n*_ and phase *ϕ*_*n*_ of each harmonic is performed at the location of the beam-splitter using low-frequency noise measurements. The calibration of each amplitude is performed by sending a single harmonic *V*_ac,*n*_ cos (2*πnft* + *ϕ*_*n*_) toward the splitter and measuring the low-frequency noise as a function of *V*_ac,*n*_. The calibration of the relative phases *ϕ*_*n*_ is more difficult and involves two-particle interferences between two harmonics at two different frequencies. As an example, to calibrate the relative phase *ϕ*_*n*_ between the first and *n*th harmonic of the signal, we generate the voltage *V*_S_(*t*) = *V*_ac,1_ cos (2*πft*) + *V*_ac,*n*_ cos (2*πnft* + *ϕ*_*n*_) at input 1 of the splitter. From the two-particle interference effect, the noise at the splitter output depends on the relative phase *ϕ*_*n*_ between the two harmonics. It is minimal (or maximal depending on the harmonics considered) when the two harmonics are in phase, allowing for an accurate calibration of the relative phase between the different harmonics. Note that for even harmonics, the two-particle interferences between the two harmonics vanish at zero bias voltage such that a small bias voltage of a few tens of microvolts needs to be added for their phase calibration. The fact that amplitude and phase of each harmonic are calibrated at the splitter location, is very important regarding the effect of Coulomb interaction during the propagation of single-electron excitations. Previous works have emphasized^53^ the importance of these effects in quantum Hall edge channels and their impact on the relaxation and decoherence^54^ of electronic excitations or on the fractionalization^55^ of these excitations. In our experimental setup, single or two-electron Lorentzian wavepackets will eventually fractionalize^26^ due to Coulomb interaction effect. However, this fractionalization will occur after the beam splitter, where we performed the tomography experiment. Indeed, these single-electron excitations result from the generation of voltage pulses propagating along the edge channels. For such types of electronics sources, which are qualitatively different from quantum dot emitters where electron emission involves the tunneling through a transmission barrier, Coulomb interaction can be taken into account by a renormalization^26^ of the amplitude and phase of each harmonic of the voltage pulse. By calibrating these amplitudes and phases at the splitter location, it means that we absorb the effect of Coulomb interaction by accommodating the amplitudes and phases of the signal to reconstruct at the splitter a Lorentzian pulse that is only limited by our calibration accuracy. This allows us to neglect Coulomb interaction effects in this experiment contrary to previous experiments^38,54^ performed with ac-driven quantum dots.

### Reconstruction of Δ*W*_S,*n*_(*ω*) from noise measurements

In this paper, we implement a reconstruction of the source Wigner distribution *W*_S_(*t*, *ω*) from the measurement of the current noise Δ*S*_*n*_ that does not rely on any assumption on the electronic state generated by the source.

First, the excess electronic distribution function Δ*W*_S,*n*=0_(*ω*) can be obtained via the derivative of the noise Δ*S*_*n*=0_ with respect to the d.c. bias *ω*_dc_ = −*eV*_dc_/ℏ applied on the probe port 2.

7 $$\Delta S_{n = 0} = 2{\mathrm{e}}^2{\cal{T}}(1 - {\cal{T}}){\int} {\frac{{d\omega }}{{2\pi }}} \left[ {\overline {\Delta W_{\mathrm{S}}} ^t\left( {1 - 2f_{{\mathrm{eq}}}\left( {\omega - \omega _{{\mathrm{DC}}}} \right)} \right)} \right]$$

8 $$\widetilde{\Delta W}_{{\mathrm{S}},0} = - \frac{\pi }{{2{\mathrm{e}}^2{\cal{T}}(1 - {\cal{T}})}}\frac{{\partial \Delta S_{n = 0}}}{{\partial \omega _{{\mathrm{DC}}}}} = {\int} d \omega \Delta W_{{\mathrm{S}},0}\left( \omega \right)\left( {\frac{{ - \partial f_{{\mathrm{eq}}}}}{{\partial \omega }}} \right)\left( {\omega - \omega _{{\mathrm{DC}}}} \right)$$

As shown by Eq. (8), the experimental signal $\widetilde {\Delta W}_{{\mathrm{S}},0}$ does not provide directly Δ*W*_S,0_ but its convolution with the thermally broadened function $\left( {\frac{{ - \partial f_{{\mathrm{eq}}}}}{{\partial \omega }}} \right)$. Knowing the electronic temperature, one can reconstruct Δ*W*_S,0_(*ω*) from the measurement of $\widetilde {\Delta W}_{{\mathrm{S}},0}$ using Bayesian deconvolution techniques presented in the next section.

We then turn to the higher-order terms, Δ*W*_S,*n*≠0_, which encode all the time dependence. They are reconstructed from the measurement of the output noise Δ*S*_*n*,*ϕ*_ as a function of the d.c. voltage *V*_DC_ and the phase *ϕ* of the a.c. voltage $V_{{\mathrm{P}}_n}(t)$ applied on the probe port. For a sinusoidal drive at frequency *nf* of small amplitude $V_{{\mathrm{P}}_n}$ on top of a d.c. drive *V*_DC_, the probe excess Wigner distribution is given by^22^ $\Delta W_{{\mathrm{P}}_n}(t,\omega ) = - \frac{{eV_{{\mathrm{P}}_n}}}{\hbar }{\mathrm{cos}}\left( {2\pi nft + \phi } \right)h_n\left( {\omega - \omega _{{\mathrm{DC}}}} \right)$, with *h*_*n*_(*ω*) = (*f*_eq_(*ω* − *nπf*) − *f*_eq_(*ω* + *nπf*))/(2*πnf*) and *ω*_DC_ = −e*V*_DC_/ℏ. Inserting this expression for the probe Wigner distribution in Eq. (3) we can reconstruct the real and imaginary parts of Δ*W*_S,*n*_:

9 $$\Re (\widetilde {\Delta W}_{{\mathrm{S}},n}) 	= \frac{h}{{8{\mathrm{e}}^3V_{{\mathrm{P}}_n}{\cal{T}}(1 - {\cal{T}})}}\left( {\Delta S_{n,\phi = 0} - \Delta S_{n,\phi = \pi }} \right) \\ 	= {\int} {\mathrm{d}} \omega \,\Re \left( {\Delta W_{{\mathrm{S}},n}\left( \omega \right)} \right){\mkern 1mu} h_n(\omega - \omega _{{\mathrm{DC}}})$$

10 $$\Im (\widetilde {\Delta W}_{{\mathrm{S}},n}) 	= \frac{h}{{8{\mathrm{e}}^3V_{{\mathrm{P}}_n}{\cal{T}}(1 - {\cal{T}})}}\left( {\Delta S_{n,\phi = \frac{\pi }{2}} - \Delta S_{n,\phi = \frac{{3\pi }}{2}}} \right) \\ 	= {\int} {\mathrm{d}} \omega \,\Im \left( {\Delta W_{{\mathrm{S}},n}\left( \omega \right)} \right)\,h_n(\omega - \omega _{{\mathrm{DC}}})$$

As in the *n* = 0 case, the experimental signal is the convolution between Δ*W*_S,*n*_ and *h*_*n*_. The real and imaginary parts of Δ*W*_S,*n*_ are thus reconstructed using deconvolution techniques (see next section). A specific difficulty arises for the *n* ≠ 0 terms, as their reconstruction process requires the accurate knowledge of amplitude and phase of the probe signals for various values of *n*. The amplitude and phase calibration of all the probe signals $V_{{\mathrm{P}}_n}(t)$ is performed similarly to the calibration of the amplitude and phase of the harmonics of the Lorentzian voltage pulses. As a result of the phase calibration, we find that, as theoretically expected for the sine and Lorentzian drives, $\Im (\widetilde {\Delta W}_{{\mathrm{S}},n}) = 0$ for all *n* and all *ω*. Note that, as mentioned above, Coulomb interaction only modifies the phase and amplitude of sinusoidal drives. As these are calibrated at the splitter location, it means that we can simply ignore Coulomb interaction effects on the probe signals. Finally, when measuring the *n* ≠ 0 harmonics, we also systematically checked the linear dependence of the output noise with the probe amplitude in order to check the validity of the linear approximation relating $\Delta W_{{\mathrm{P}}_n}(t,\omega )$ to $V_{{\mathrm{P}}_n}(t)$.

### Bayesian deconvolution method

The relation between Δ*W*_S,*n*_(*ω*) and $\widetilde {\Delta W}_{{\mathrm{S}},n}(\omega )$ is given by the convolution product, see Eqs. (8)–(10):

11 $$\widetilde {\Delta W}_{{\mathrm{S}},n}(\omega ) = \left( {h_n \ast \Delta W_{{\mathrm{S}},n}} \right)(\omega )$$

12 $$= {\int} d\omega{\prime} h_n(\omega{\prime} - \omega )\Delta W_{{\mathrm{S}},n}(\omega{\prime} )$$

In order to estimate Δ*W*_S,*n*_(*ω*) based on $\widetilde {\Delta W}_{{\mathrm{S}},n}(\omega )$, we need to implement a deconvolution algorithm. Since deconvolution is an ill-posed problem, simply performing a division in Fourier space leads to an estimation, which is not robust to measurement errors. The sensibility to errors, due to lost information, corresponds to zero or close to zero values of the Fourier transform of *h*_*n*_. In order to find a more robust estimation with correct physical properties, we propose to add appropriate prior information on Δ*W*_S,*n*_(*ω*) thanks to a Bayesian framework^56–58^.

The discretized forward model for convolution (12) can be expressed as

13 $$\widetilde {{\mathbf{\Delta W}}}_{{\mathrm{S}},n} = {\mathbf{H}}_n.{\mathbf{\Delta W}}_{{\mathrm{S}},n} + {\mathbf{N}}_n,$$

where bold characters stand for vectors and matrices resulting from discretization. $\widetilde {\Delta W}_{{\mathrm{S}},n}$ is the vector of data points, **H**_*n*_ is the convolution matrix, and **ΔW**_S,*n*_ is the unknown quantity we are looking for. The term **N**_*n*_ is added to take account for all the errors (measurement and discretization). It is modeled as Gaussian random vector, with a known covariance matrix **V**_e_ with diagonal elements *V*_e,*i*_ estimated, thanks to repeated experiments. This gives the expression of the probability distribution of $\widetilde {\Delta W}_{{\mathrm{S}},n}$ knowing **ΔW**_S,*n*_ and **V**_e_, which is called the likelihood

14 $$p\left( {\widetilde {{\mathbf{\Delta W}}}_{{\mathrm{S}},n}\left| {{\mathbf{\Delta W}}_{{\mathrm{S}},n},{\mathbf{V}}_{\mathrm{e}}} \right.} \right) \propto exp\left( { - \frac{1}{2}\left\| {\widetilde {{\mathbf{\Delta W}}}_{{\mathrm{S}},n} - {\mathbf{H}}_n.{\mathbf{\Delta W}}_{{\mathrm{S}},n}} \right\|_{{\mathbf{V}}_{\mathrm{e}}}^2} \right)$$

where $\left\| {\mathbf{x}} \right\|_{{\mathbf{V}}_{\mathrm{e}}}^2 = \mathop {\sum}\nolimits_i \frac{{x_i^2}}{{v_{{\mathrm{e}}_i}}}$. Finding the argument that maximizes the likelihood, is equivalent to performing a division in Fourier space, since the convolution matrix **H**_*n*_ is diagonal in the Fourier basis. This argument is dominated by ${\mathbf{H}}_n^{ - 1}{\mathbf{N}}_n$ terms.

In the Bayesian framework, by adding a prior information, we want to enforce physical properties such that Δ*W*_S,*n*_(*ω*) tends to zero when |*ω*| increases. For this purpose, we assign a Gaussian prior distribution on **ΔW**_S,*n*_:

15 $$p\left( {{\mathbf{\Delta W}}_{{\mathrm{S}},n}|{\mathbf{V}}_{\mathrm{f}}} \right) \propto exp\left( { - \frac{1}{2}\left\| {{\mathbf{\Delta W}}_{{\mathrm{S}},n}} \right\|_{{\mathbf{V}}_{\mathrm{f}}}^2} \right).$$

For variances **V**_f_, we use the expression

16 $$V_{\mathrm{f}}(\omega ) = v_{\mathrm{f}}exp\left( { - \frac{{\omega ^2}}{{w^2}}} \right),$$

where *v*_f_ and *w* are parameters tuned to enforce limit condition when |*ω*| increases (the influence of the parameters *w* and *v*_f_ on the deconvoluted signal are presented in the Supplementary Note 4).

Applying Bayes’rule, the posterior probability distribution of **ΔW**_S,*n*_ combines likelihood (14) and prior distribution (15):

17 $$p\left( {{\mathbf{\Delta W}}_{{\mathrm{S}},n}|\widetilde {{\mathbf{\Delta W}}}_{{\mathrm{S}},n},{\mathbf{V}}_{\mathrm{e}},{\mathbf{V}}_{\mathrm{f}}} \right) = \frac{{p\left( {\widetilde {{\mathbf{\Delta W}}}_{{\mathrm{S}},n}|{\mathbf{\Delta W}}_{{\mathrm{S}},n},{\mathbf{V}}_{\mathrm{e}}} \right)p\left( {{\mathbf{\Delta W}}_{{\mathrm{S}},n}|{\mathbf{V}}_{\mathrm{f}}} \right)}}{{p\left( {\widetilde {{\mathbf{\Delta W}}}_{{\mathrm{S}},n}|{\mathbf{V}}_{\mathrm{e}},{\mathbf{V}}_{\mathrm{f}}} \right)}}{\kern 1pt} .$$

The argument that maximizes this posterior distribution (17), is the most likely estimate of **ΔW**_S,*n*_ knowing both the measurement results $\widetilde {{\mathbf{\Delta W}}}_{{\mathrm{S}},n}$, **V**_e_, and prior information encoded in **V**_f_. Indeed, this maximum a posteriori (MAP), which also in this case is the posterior mean, is robust to errors **N**_*n*_. Because of model evidence, the term in the denominator of (17), $p\left( {\widetilde {\Delta W}_{{\mathrm{S}},n}|{\mathbf{V}}_{\mathrm{e}},{\mathbf{V}}_{\mathrm{f}}} \right)$ does not depend on **ΔW**_S,*n*_, MAP estimate becomes equivalent to the minimization of the criterion:

18 $$J\left( {{\mathbf{\Delta W}}_{{\mathrm{S}},n}} \right) = \frac{1}{2}\left\| {\widetilde {{\mathbf{\Delta W}}}_{{\mathrm{S}},n} - {\mathbf{H}}_n.{\mathbf{\Delta W}}_{{\mathrm{S}},n}} \right\|_{{\mathbf{V}}_{\mathrm{e}}}^2 + \frac{1}{2}\left\| {{\mathbf{\Delta W}}_{{\mathrm{S}},n}} \right\|_{{\mathbf{V}}_{\mathrm{f}}}^2.$$

The estimated **ΔW**_S,*n*_ has to comply with a box constraint given by Pauli exclusion principle and Cauchy–Schwartz inequality^22^. Consequently, the implemented algorithm in Box 1 looks for a minimum of criterion (18) inside the box constraint, thanks to a Projected Gradient Descent method^59^. In this algorithm, *M*_*n*_(*ω*) denotes Cauchy–Schwartz inequality bounds ∀*n*,*ω* and is explicitly given in ref. ^22^.

Box 1 Detailed deconvolution algorithm Compute Cauchy–Schwartz bounds *M*_*n*_(*ω*)

Choose amplitude *v*_f_ and width *w* of prior (16) for *V*_f_(*ω*)

Compute the minimum of criterion (18)

$${\mathbf{\Delta W}}_{{\mathrm{S}},n} = \left( {{\mathbf{H}}^{\rm{T}}{\mathbf{V}}_{\mathrm{e}}^{ - 1}{\mathbf{H}} + {\mathbf{V}}_{\mathrm{f}}^{ - 1}} \right)^{ - 1}{\mathbf{H}}^{\rm{T}}{\mathbf{V}}_{\mathrm{e}}^{ - 1}\widetilde {{\mathbf{\Delta W}}}_{{\mathrm{S}},n}$$

Project the solution inside the box given by Cauchy–Schwartz bounds:

Δ*W*_S,*n*_(*ω*): = min(Δ*W*_S,*n*_(*ω*),*M*_*n*_(*ω*))

and Δ*W*_S,*n*_(*ω*): = max(Δ*W*_S,*n*_(*ω*), −*M*_*n*_(*ω*))

**repeat**

Compute the gradient of criterion (18)

$$\nabla \left( {{\mathbf{\Delta W}}_{{\mathrm{S}},n}} \right) = - {\mathbf{H}}^{\rm{T}}{\mathbf{V}}_{\mathrm{e}}^{ - 1}\left( {\widetilde {{\mathbf{\Delta W}}}_{{\mathrm{S}},n} - {\mathbf{H\Delta W}}_{{\mathrm{S}},n}} \right) + {\mathbf{V}}_{\mathrm{f}}^{ - 1}{\mathbf{\Delta W}}_{{\mathrm{S}},n}$$

Project the gradient **P▽**(**ΔW**_S,*n*_) to stay in the box constraint

**if** |Δ*W*_S,*n*_(*ω*)| ≥ *M*_*n*_(*ω*) and Δ*W*_S,*n*_(*ω*)*∇(Δ*W*_S,*n*_)(*ω*) ≤ 0 **then**

P∇(Δ*W*_S,*n*_)(*ω*) = 0

**else**

P∇(Δ*W*_S,*n*_)(*ω*) = ∇(Δ*W*_S,*n*_)(*ω*)

**end if**

Compute the furthest displacement *d*_∞_ in the box along **P▽**(**ΔW**_S,*n*_) direction

**For all** P∇(Δ*W*_S,*n*_)(*ω*) ≠ 0 **do**

$$d_\infty : = {\mathrm{min}}\left( {\frac{{M_n(\omega ) - \Delta W_{{\mathrm{S}},n}(\omega )}}{{{\mathrm{P}}\nabla \left( {\Delta W_{{\mathrm{S}},n}} \right)(\omega )}},d_\infty } \right)$$

**end for**

Compute the optimum displacement *d*_0_ along **P∇**(**ΔW**_S,*n*_) direction

$$d_0 = \left\| {{\mathrm{P}}\nabla \left( {{\mathbf{\Delta W}}_{{\mathrm{S}},n}} \right)} \right\|^{ - 2}\left( {\left\| {{\mathbf{H}}{\mathrm{P}}\nabla \left( {{\mathbf{\Delta W}}_{{\mathrm{S}},n}} \right)} \right\|_{{\mathbf{V}}_{\mathrm{e}}}^2} \right. + \left. {\left\| {{\mathrm{P}}\nabla \left( {{\mathbf{\Delta W}}_{{\mathrm{S}},n}} \right)} \right\|_{{\mathbf{V}}_{\mathrm{f}}}^2} \right)$$

Compute one projected descent gradient step

**ΔW**_S,*n*_: = **ΔW**_S,*n*_ − min(*d*_0_, *d*_∞_)P∇(**ΔW**_S,*n*_)

**until** $- ln\left( {p\left( {{\mathbf{\Delta W}}_{{\mathrm{S}},n}\left| {\widetilde {{\mathbf{\Delta W}}}_{{\mathrm{S}},n},{\mathbf{V}}_{\mathrm{e}},{\mathbf{V}}_{\mathrm{f}}} \right.} \right)} \right)$ is minimized

### Electron and hole wavefunction extraction

The extraction of electron and hole wavefunctions from the experimental data for Δ*W*_S_(*t*, *ω*) relies on an algorithm that recasts any excess *T*-periodic single-electron coherence under the form given by Eq. (2) of the article. The algorithm is a generalization of the Kahrunen–Loève analysis^60^ to electron quantum optics. It is based on an exact diagonalization of the projections of the single-electron coherence (represented by Δ_0_*W*_S_(*t*, *ω*)) onto the electronic and hole quadrants with respect to the reference chemical potential (here *μ* = 0). As explained in ref. ^22^, these projections are defined by decomposing the space of a single-particle state into positive (for electrons) and negative energy (for holes) states.

In practice, they are obtained through the following procedure: after deconvolution, the experimental data come as a finite set of real values Δ*W*_S,*n*_(*ω*_*k*_) where *ω*_*k*_ are the discretized values of *ω* and *n* = 0, ±1, ±2,…,±5. First, we add the thermal excess *f*_*eq*_(*ω*) − Θ(−*ω*) of the equilibrium Fermi–Dirac distribution at temperature *T*_el_ to Δ*W*_S,*n*=0_(*ω*) to obtain the experimental data set for Δ_0_*W*_S_(*ω*). Then, in order to extract a square matrix for the exact diagonalization, the next step is to interpolate the data on a grid well suited to the electronic and hole quadrants. These two quadrants are defined in the frequency domain as corresponding to the sectors, where purely electronic (resp. purely hole) excitations contribute to Δ*W*_S,*n*_(*ω*). For a periodically driven source, they correspond to *ω* ≥ |*n*|*πf* for the electron quadrant and to *ω* ≤ −|*n*|*πf* for the hole quadrant^22^. For each *n*, the data set Δ_0_*W*_S,*n*_(*ω*_*k*_) is first interpolated using cubic splines to infer a new data set on a grid adapted to the electronic and hole quadrants (i.e., such that this grid intersects the boundaries *ω* ± *nπf* = 0 of the electronic and hole quadrants). This new data grid has a discretization step *δω* such that $\hbar {\kern 1pt} \delta \omega \simeq 0.19\,{\mathrm{\mu eV}}$. This data set is then used to build the matrices corresponding to the projections on the electron and hole quadrants of this interpolated data for Δ_0_*W*_S,*n*_(*ω*).

Due to time periodicity of the single-electron Wigner function Δ_0_*W*_S_(*t*,*ω*) = Δ_0_*W*_S_(*t* + *T*, *ω*), diagonalizing these two projections onto the electron and hole quadrants leads to electronic (*α* = e) and hole (*α* = h) probability spectral bands $g_i^{(\alpha \alpha )}(\nu )$ (*i* being a band index) depending on quasi-pulsation interval 0 ≤ *ν* ≤ 2*πf* associated with the time period *T*. The corresponding eigenvectors are the Floquet version of Bloch waves of solid-state physics.

Then, the electron and hole wavefunctions $\varphi _{l,i}^{(\alpha )}$ generated at each time period^14,61^ are the analogous of the Wannier functions^62^. They consist of normalized single-particle wavepackets such that

19 $$\varphi _{l,i}^{(\alpha )}(t) = \varphi _i^{(\alpha )}(t - lT)$$

20 $$\langle \varphi _{l{\prime},i{\prime}}^{(\alpha{\prime})}|\varphi _{l,i}^{(\alpha )}\rangle = \delta _{i,i{\prime}}\,\delta _{\alpha ,\alpha{\prime}}\,\delta _{l,l{\prime}}.$$

where *α* denotes the electron or hole label and *i* the band index. These electron and hole wavefunctions are therefore very well suited to describe the excitations generated by time-periodic electron beams. In solid-state physics^63^, the Wannier wavefunctions are not uniquely defined since one can impose an arbitrary quasi-momentum dependent phase in front of each Bloch wave. Here, the same problem is present, and exactly as in solid-state physics, this ambiguity is lifted by minimizing their time spreading. This provides us electronic and hole atoms of signals that are maximally localized in the time domain. Finally, the electron coherence $g_i^{({\mathrm{ee}})}(l)$ between electronic atoms of signals translated by $l \in {\Bbb Z}^ \ast$ time periods as well as the hole coherence $g_i^{({\mathrm{hh}})}(l)$ can be obtained from the probability spectra through Fourier transform. For $l \in {\Bbb Z}$

21 $$g_i^{(\alpha \alpha )}(l) = {\int}_{\!\!\!0}^{2\pi f} {e^{i\nu l/f}} g_i^{(\alpha )}(\nu )\frac{{d\nu }}{{2\pi f}}$$

Note that there is no electronic coherence between atoms of a signal of the same type of excitation, but with a different band index. The electron/hole coherences $g_{i,i{\prime}}^{({\mathrm{eh}})}(l)$ are defined as the single-electron coherence between the electronic atom of signal $\varphi _{i,l}^{({\mathrm{e}})}$ and the hole atom of signal $\varphi _{i{\prime},0}^{({\mathrm{h}})}$:

22 $$g_{i,i{\prime}}^{({\mathrm{eh}})}(l) = {\mathrm{Tr}}\left( {\psi [\varphi _{l,i}^{({\mathrm{e}})}]{\mkern 1mu} \rho {\mkern 1mu} \psi ^\dagger [\varphi _{0,i{\prime}}^{({\mathrm{h}})}]} \right)$$

23 $$= {\int} {\mathrm{d}} t{\mathrm{d}}t{\prime}\varphi _{l,i}^{({\mathrm{e}})}(t)^ \ast \,\Delta _0{\cal{G}}(t,t{\prime})\,\varphi _{0,i{\prime}}^{({\mathrm{h}})}(t{\prime}).$$

where $l \in {\Bbb Z}$, *i*, and *i*′ are possibly different. It is obtained from the electronic Wigner function using the explicit numerical data for the electronic and hole wavefunctions.

### Purity indicator

The general expression of the state purity is beyond the scope of this paper. We focus here on the two limiting cases considered in this paper: the sinusoidal drives where one electron and one hole wavefunction are generated and the periodic train of single-electron Lorentzian pulses, where two or three electronic wavefunctions need to be considered (the probability for hole emission can be neglected).

Let us consider first the sinusoidal drive case with only one electron and one hole branch so that the branch index *i* can be dropped out. Time periodicity implies that only coherences between Floquet–Bloch eigenvectors with the same quasi-pulsation do not vanish. As only one electron and one hole wavepackets are emitted, we can therefore consider, at each given quasi-pulsation 0 ≤ *ν* < 2*πf*, the reduced density matrix *ρ*_eh_ in the occupation number basis of the electron and hole states: |*n*_e_*n*_h_〉. *n*_e_ = 0 or 1 and *n*_h_ = 0 or 1 are the occupation numbers of the corresponding single-particle states. As a result of the superselection rule that forbids quantum superposition between states with fermion numbers of different parity^64,65^, *ρ*_eh_ corresponds to a pure state in three situations: either the electronic and hole levels are both filled (state |11〉), or both empty (state |00〉), or populated in a coherent way (state *u*|01〉 + *v*|10〉 with |*u*|^2^ + |*v*|^2^ = 1). Any deviation from purity thus reflects incoherent electron/hole processes. The purity indicator, which is defined as ${\mathrm{Tr}}(\rho _{{\mathrm{eh}}}^2)$, is a good quantity for measuring the weight of coherent processes:

24 $${\mathrm{Tr}}(\rho _{{\mathrm{eh}}}^2) = 1 - 2A(\nu )(1 - A(\nu )) - 2B(\nu )(1 - B(\nu ))$$

where

25 $$A(\nu ) = g^{({\mathrm{ee}})}(\nu )(1 - g^{({\mathrm{hh}})}(\nu )) - |g^{({\mathrm{eh}})}(\nu )|^2$$

26 $$B(\nu ) = g^{({\mathrm{hh}})}(\nu )(1 - g^{({\mathrm{ee}})}(\nu )) - |g^{({\mathrm{eh}})}(\nu )|^2$$

are computed in terms of the eigenvalues *g*^(ee)^(*ν*) and *g*^(hh)^(*ν*) obtained from our diagonalization algorithm and of the corresponding electron/hole coherences *g*^(eh)^(*ν*).

Let us now discuss all the reduced density matrices *ρ*_eh_ for all 0≤*ν* < 2*πf*. When Wick’s theorem is valid, there are no correlations between different quasi-pulsations *ν*_1_ ≠ *ν*_2_. Consequently, we can take the infinite-dimensional product over all the Floquet–Bloch pairs of electron and hole modes for 0 ≤ *ν* < 2*πf* and take the trace of its square, which is a formal infinite product over all 0 ≤ *ν* < 2*πf*. This quantity has to be regularized in the infrared by discretizing the quasi energies *ν*_*n*_=2*πfn*/*N* for *n* = 0…*N* − 1 and taking the 1/*N*th power of the result. This procedure leads to the resulting quantity

27 $${\Bbb P} = {\mathrm{exp}}\left[ {{\int}_{\!\!\!0}^{2\pi f} ln\left( {1 - 2A(\nu )(1 - A(\nu )) - 2B(\nu )(1 - B(\nu ))} \right)\frac{{d\nu }}{{2\pi f}}} \right]$$

which is equal to unity if and only if the many-body state is pure and obtained from a Fermi sea vacuum by adding on top of it coherent superposition of electron/hole pairs. When there are incoherent processes such as in the case of nonzero temperature, ${\Bbb P} < 1$. In the present situation, the condition *A(ν*) = *B(ν*) = 0, which ensures unit purity, corresponds to |*g*^(eh)^(*ν*)|^2^ = *g*^(ee)^(*ν*)(1 − *g*^(hh)^(*ν*)) = *g*^(hh)^(*ν*)(1 − *g*^(ee)^(*ν*)), which implies that *g*^(ee)^(*ν*) = *g*^(hh)^*(ν*). Then, the condition on *g*^(eh)^*(ν*) ensures that, for each quasi-pulsation *ν*, we have acted on the Fermi sea |*F*〉 through the coherent sum of the identity operator, and of the elementary electron/hole pair creation operator that is the product of a creation operator for the electron single-particle state and of a destruction operator for the hole single-particle state. This is equivalent to putting each quasi-particle which, in |*F*〉, is in the hole state $\varphi _\nu ^{({\mathrm{h}})}$, into the linear combination $u(\nu )\varphi _\nu ^{({\mathrm{h}})} + v(\nu )\varphi _\nu ^{({\mathrm{e}})}$. The resulting many-body state is then pure and of the form

28 $$\left| \Psi \right\rangle = \mathop {\prod}\limits_{0 \le \nu < 2\pi f} {\left( {u(\nu ) + v(\nu )\psi ^\dagger [\varphi _\nu ^{({\mathrm{e}})}]\psi [\varphi _\nu ^{({\mathrm{h}})}]} \right)} |F\rangle .$$

This specific form was also obtained in ref. ^30^, which considered a conductor at zero temperature described by a single-particle time-dependent scattering matrix. It reduces to the form given by Vanevic et al.^29^ in the case where the Floquet–Bloch spectrum is flat as a function of *ν* (in which case there are no interperiod electronic and hole coherences).

Equation (27) for the purity can be adapted to the Lorentzian case we study in the paper. Three electronic bands need to be considered at most for the Lorentzian pulse carrying two electrons. These bands are not coupled in our specific experimental situation (the term coupling the two bands are the electron–hole coherences $g_{ij}^{({\mathrm{eh}})}$, which we measure to be negligible). In this case, the extension of Eq. (27) is straightforward:

29 $${\Bbb P} = {\Bbb P}_1 \times {\Bbb P}_2 \times {\Bbb P}_3$$

30 $${\Bbb P}_i = {\mathrm{exp}}\left[ {{\int}_{0}^{2\pi f} {\ln } \left( {1 - 2g_i^{({\mathrm{ee}})}(\nu )(1 - g_i^{({\mathrm{ee}})}(\nu ))} \right)\frac{{d\nu }}{{2\pi f}}} \right]$$

where we have used the simplified expressions of $A_i(\nu ) = g_i^{({\mathrm{ee}})}(\nu )$ and *B*_*i*_(*ν*) = 0 in the case where $g_i^{({\mathrm{hh}})}(\nu ) \approx g_{ij}^{({\mathrm{eh}})}(\nu ) \approx 0$. The pure state ${\Bbb P} = {\Bbb P}_i = 1$ is only recovered for $g_i^{({\mathrm{ee}})}(\nu ) = 0$ or 1. In our experimental situation for the single-electron Lorentzian pulse, two wavefunctions are emitted with probabilities smaller than 1 and we find ${\Bbb P} = 0.75$, showing that the generated state is a mixture of two single-electron wavefunctions. For the two-electron Lorentzian pulse, we find a slightly smaller purity ${\Bbb P} = 0.68$.

## Supplementary information

Supplementary Information

## Data Availability

The data that support the findings of this study are available from the corresponding author upon reasonable request.
