# Supplementary material for: Quantum tomography of electrical currents
Source: Nat Commun. 2019 Jul 29;10:3379. doi: 10.1038/s41467-019-11369-5 (PMC6662746; doi:10.1038/s41467-019-11369-5)
Supplement: Supplementary file 1 — Supplementary Information [file 41467_2019_11369_MOESM1_ESM.pdf]

Quantum tomography of electrical currents: supplementary information

R. Bisognin[1†], A. Marguerite[1†], B. Roussel[2,5], M. Kumar[1], C. Cabart[2], C. Chapdelaine[4], A. Mohammad-Djafari[4], J.-M. Berroir[1], E. Bocquillon[1], B. Plaçais[1], A. Cavanna[3], U. Gennser[3], Y. Jin[3], P. Degiovanni[2], and G. Fève[1*]

[1] Laboratoire de Physique de l'Ecole normale supérieure, ENS, Université PSL, CNRS, Sorbonne Université, Université Paris-Diderot, Sorbonne Paris Cité, Paris, France

[2] Univ Lyon, Ens de Lyon, Université Claude Bernard Lyon 1, CNRS, Laboratoire de Physique, F-69342 Lyon, France.

[3] Centre de Nanosciences et de Nanotechnologies (C2N), CNRS, Univ. Paris-Sud, Université Paris-Saclay, 91120 Palaiseau, France.

[4] Laboratoire des signaux et systèmes, CNRS, Centrale-Supélec- Université Paris-Saclay.

[5] European Space Agency - Advanced Concepts Team, ESTEC, Keplerlaan 1, 2201 AZ Noordwijk, The Netherlands.

[*] To whom correspondence should be addressed; E-mail: feve@lpa.ens.fr.

[†] These authors contributed equally.

SUPPLEMENTARY NOTE 1. SIMULATIONS USING FLOQUET SCATTERING THEORY

The numerical simulations discussed in the paper are performed by computing the electronic coherence $\mathcal{G}(t,t')$ generated by applying a time-dependent voltage $V(t)$ to the edge channel. We start from the expression of $\mathcal{G}(t,t')$ when a single voltage pulse is generated[1]:

$$\mathcal{G}(t,t') = e^{\frac{ie}{\hbar}\int_{t'}^{t} V(\tau)d\tau}\, \mathcal{G}_{\mu}(t,t') \tag{1}$$

where $\mathcal{G}_{\mu}(t,t')$ is the equilibrium electronic coherence of an edge channel at temperature T_{el} and chemical potential μ:

$$\mathcal{G}_{\mu}(t,t') = \int \frac{d\omega}{2\pi} f_{\mu}(\omega) e^{-i\omega(t-t')} \tag{2}$$

In the case of the periodic generation of voltage pulses, on needs to decompose the time dependent voltage $V(t)$ into its ac and dc parts, as the dc part induces a shift of the chemical potential of the channel:

$$V(t) = V_{\text{ac}}(t) - \Delta\mu/e \tag{3}$$

$$\mathcal{G}(t,t') = e^{\frac{ie}{\hbar}\int_{t'}^{t} V_{\text{ac}}(\tau)d\tau}\, \mathcal{G}_{\mu+\Delta\mu}(t,t') \tag{4}$$

Using the periodicity of the ac drive, this expression is more easily computed in Fourier space:

$$\mathcal{G}(t,t') = \sum_{n=-\infty}^{+\infty} e^{-i2\pi n f\bar{t}} \int \frac{d\omega}{2\pi}\, \mathcal{G}_n(\omega) e^{-i\omega(t-t')} \text{ with } \bar{t} = \frac{t+t'}{2} \tag{5}$$

$$\mathcal{G}_n(\omega) = \sum_{l} c_{n+l} c_l^{*}\, f_{\mu+\Delta\mu}\big(\omega - (n+2l)\pi f\big) \tag{6}$$

$$e^{\frac{ie}{\hbar}\int_{0}^{t} V_{\text{ac}}(\tau)d\tau} = \sum_{l} c_l\, e^{-i2\pi l f t} \tag{7}$$

Using these expressions, the Wigner distribution for any kind of periodic drive can be numerically computed. In the next sections, we compute the Wigner distributions for $q = -e$ and $q = -2e$ periodic Lorentzian pulses and extract the emitted wavefunctions from these numerically computed Wigner distributions. It allows us to compare these calculations with our experimental observations and to predict what are the effects of finite temperature on the generated electronic states in a wide range of temperatures.

SUPPLEMENTARY NOTE 2. TEMPERATURE EFFECTS ON LORENTZIAN PULSES: $q = -e$

We have plotted on Fig.1.a the numerical computations of the Wigner distribution for periodic Lorentzian drives, $V(t) = \sum_l -\frac{V_0}{1+(t-lT)^2/\tau^2}$, with $\tau = 40$ ps and V_0 chosen such that each pulse carries a single electron, $q = -e$. The three temperatures are $T_{\text{el}} = 0.01$ K (corresponding to $k_B T_{\text{el}}/(hf) = 0.05$), $T_{\text{el}} = 0.05$ K ($k_B T_{\text{el}}/(hf) = 0.25$) and $T_{\text{el}} = 0.1$ K ($k_B T_{\text{el}}/(hf) = 0.5$). The influence of the temperature on the Wigner distribution $W(t,\omega)$ is important: at fixed width of the lorentzian pulse τ, the non-classical features $W(t,\omega) < 0$ and $W(t,\omega) > 1$ are progressively suppressed as temperature increases. They are still visible for $T_{\text{el}} = 0.05$ K but not anymore for $T_{\text{el}} = 0.1$ K. This corresponds to the suppression of the steplike features on the energy distribution which are smoothed by increasing temperature. On the contrary, the electrical current is unaffected by the temperature, it is always given by $I(t) = \frac{e^2}{h} V(t)$.

Having numerically computed the Wigner distribution $W(t,\omega)$ for various temperatures, we can run the diagonalization algorithm and extract the relevant electronic wavefunctions $\varphi_i^{(\text{e})}$ and their emission probabilities. $\varphi_1^{(\text{e})}$ and $\varphi_2^{(\text{e})}$ extracted from the Wigner distribution at $T_{\text{el}} = 0.05$ K are plotted on Fig.1.b. They are very similar to our experimental observations presented in the main manuscript. Surprisingly, the influence of the temperature on the nature of the wavefunctions is very small. Indeed, the overlap between $\varphi_1^{(\text{e})}$ extracted for $T_{\text{el}} = 0.01$ K and $\varphi_1^{(\text{e})}$ extracted for $T_{\text{el}} = 0.1$ K is very large (≈ 0.99). $\varphi_1^{(\text{e})}$ matches the first wavefunction for periodic trains of Lorentzian pulses expected at zero temperature: $\varphi_1^{(\text{e})} \approx \varphi_{\text{L},n=1}$. On the contrary, finite temperature has a very strong influence on the emission probabilities. The evolution of the emission probabilities $p_i^{(\text{e})}$ for the first three electronic wavefunctions $\varphi_i^{(\text{e})}$ and the emission probability of the first hole wavefunction $p_1^{(\text{h})}$ are plotted on Fig.2 for various temperatures and various width τ of the Lorentzian pulse in normalized units. At $T_{\text{el}} = 0$ K, as expected, only $\varphi_1^{(\text{e})}$ is emitted with $p_1^{(\text{e})} = 1$ independently of τ. When the temperature is increased (depending on the ratio $k_B T_{\text{el}}/(hf)$) more and more electronic and hole wavefunctions are emitted with probabilities $p_i^{(\alpha)} < 1$ corresponding to a more and more mixed state. Interestingly, at fixed temperature, generating narrower and narrower pulses (increasing the ratio T/τ) to an increase of the probability to emit the first wavefunction $p_1^{(\text{e})}$ (and the second wavefunction $p_2^{(\text{e})}$) as well, but with the price of an increasing probability to emit a hole: $p_1^{(\text{h})}$.

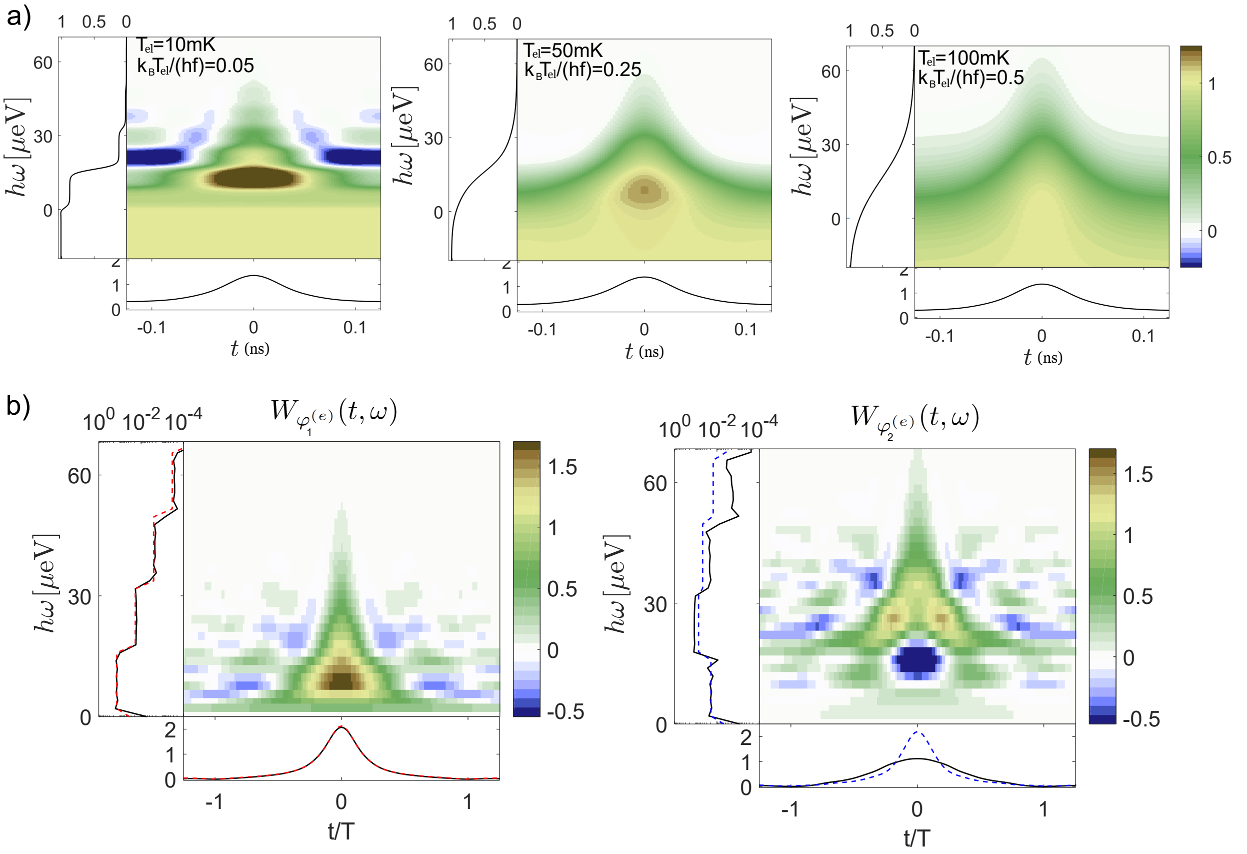

SUPP. FIG. 1. **a** Wigner distribution $W(t,\omega)$ calculated using Floquet scattering theory for periodic Lorentzian pulses carrying a charge $q=-e$ with $\tau=40$ ps and $T_{\rm el}=0.01$, 0.05 and 0.1 K. **b** $\varphi_1^{\rm (e)}$ and $\varphi_2^{\rm (e)}$ extracted from $W(t,\omega)$. The red dashed lines represent the Lorentzian wavefunctions $\varphi_{{\rm L},n=1}$ and the blued dashed lines $\varphi_{{\rm L},n=2}$.

SUPPLEMENTARY NOTE 3. TEMPERATURE EFFECTS ON LORENTZIAN PULSES: $q=-2e$

The same numerical analysis can be carried for the $q=-2e$ Lorentzian pulse. The Wigner distributions computed for the three temperatures $T_{\rm el}=0.01$ K, $T_{\rm el}=0.05$ K and $T_{\rm el}=0.1$ K are plotted on Fig.3. The effect of temperature is similar to the $q=-e$ case. The non-classical features on the Wigner distribution are fully suppressed at $T_{\rm el}=0.1$ K, as well as the steps in the electronic distribution function $f(\omega)$. However, the current $I(t)$ is unaffected by increasing temperature.

As in the $q=-e$ case, finite temperature has a strong influence on the emission probabilities $p_i^{\rm (e)}$ and $p_i^{\rm (h)}$ which are plotted on Fig.4. At $T_{\rm el}=0$, as expected, two wavefunctions are emit-

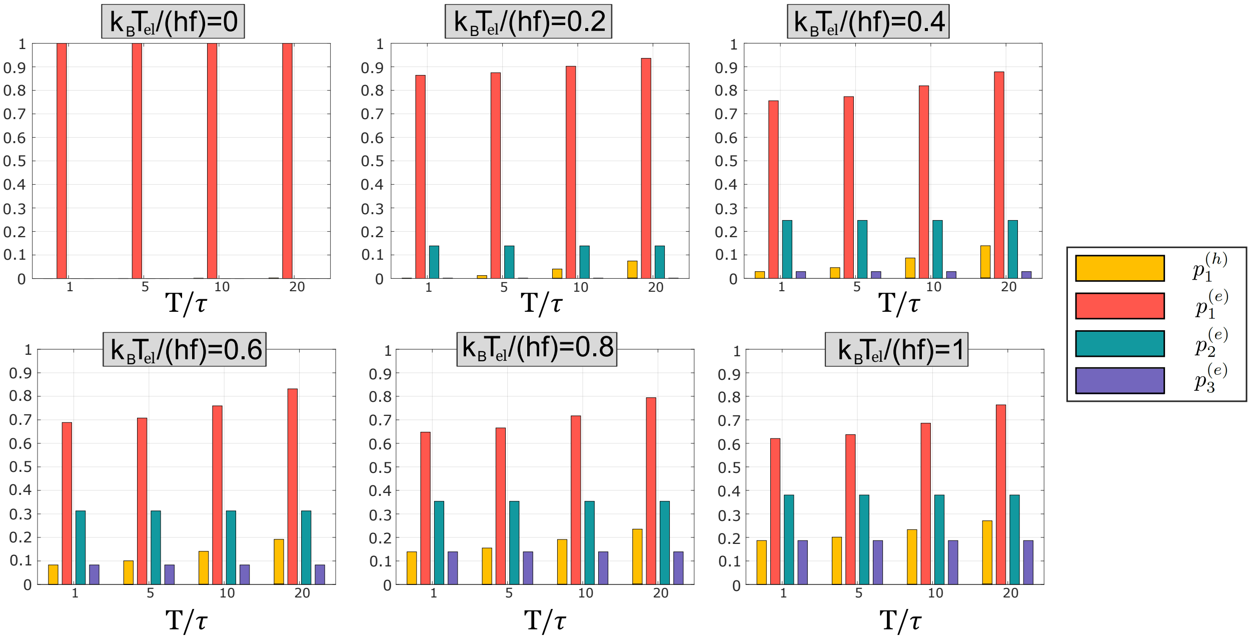

SUPP. FIG. 2. Evolution of electron and hole emission probabilities with temperature and pulse width in normalized units $k_B T_{\text{el}}/(hf)$ and T/τ.

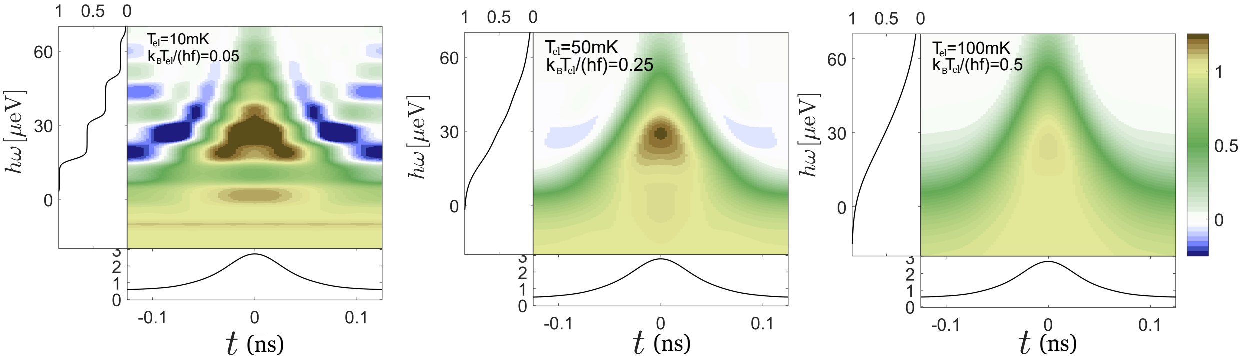

SUPP. FIG. 3. Wigner distribution $W(t, \omega)$ calculated using Floquet scattering theory for periodic Lorentzian pulses carrying two charges $q = -2e$ with $\tau = 40$ ps and $T_{\text{el}} = 0.01, 0.05$ and 0.1 K.

ted with probabilities $p_1^{(\text{e})} = p_2^{(\text{e})} = 1$ and no hole excitation is generated $p_1^{(\text{h})} = 0$. When the temperature is increased, $p_2^{(\text{e})}$ and eventually even $p_1^{(\text{e})}$ decrease from 1 and additional states are generated with probabilities $p_{i>2}^{(\text{e})} < 1$ as the state evolves from a pure state to a statistical mixture. Additionally, the probability to generate a hole excitation also increases with temperature. For shorter and shorter pulses (increasing values of the ratio T/τ), the probability to emit the second wavefunctions $p_2^{(\text{e})}$ increases towards 1 while the probability to emit a hole $p_1^{(\text{h})}$ also increases.

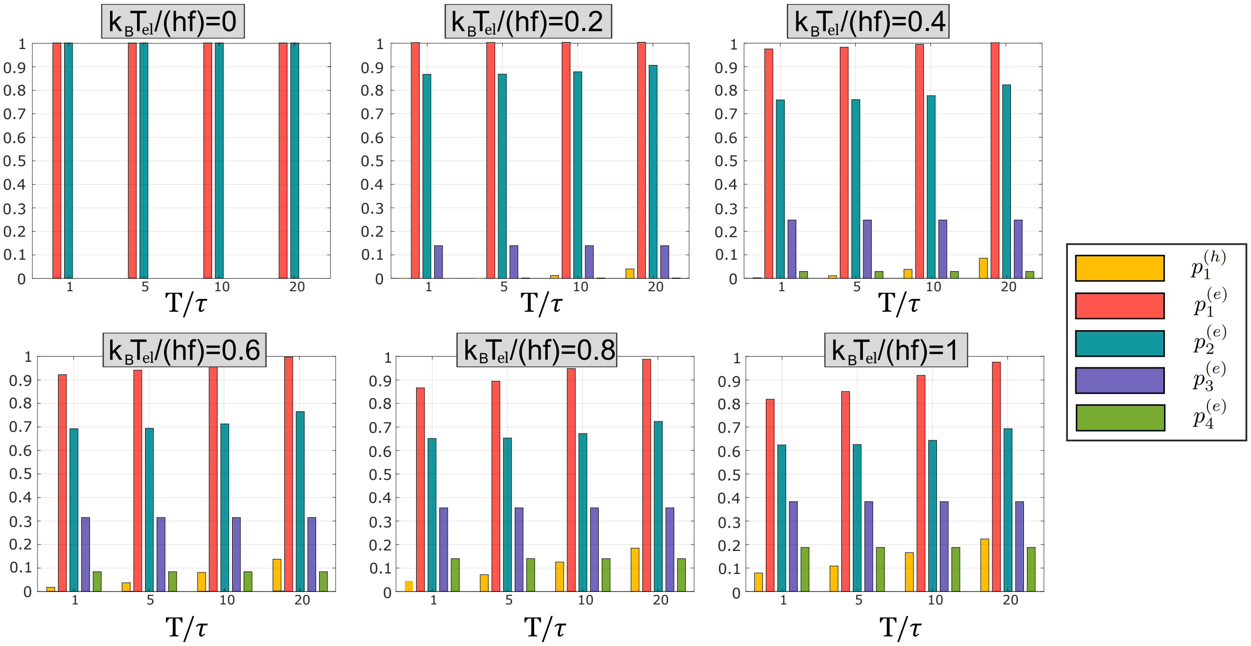

SUPP. FIG. 4. Evolution of electron and hole emission probabilities with temperature and pulse width in normalized units $k_B T_{\text{el}}/(hf)$ and T/τ.

The effect of finite temperature on the nature of the wavefunctions is more subtle for $q = -2e$ compared to the $q = -e$ case. The two generated wavefunctions $\varphi_1^{(\text{e})}$ and $\varphi_2^{(\text{e})}$ extracted from $W(t,\omega)$ in the low temperature case $k_B T_{\text{el}}/(hf) \ll 1$ are plotted on Fig.5. They do not correspond to the expected wavefunctions $\varphi_{\text{L},n=1}$ (plotted in red dashed line) and $\varphi_{\text{L},n=2}$ (plotted in blue dashed line). $\varphi_1^{(\text{e})}$ and $\varphi_2^{(\text{e})}$ are symmetric with respect to $t = 0$. They are more localized in time than $\varphi_{\text{L},n=1}$ and $\varphi_{\text{L},n=2}$, this is a consequence of our extraction algorithm which looks for the wavefunctions which are maximally localized in time (see Methods section).

For $k_B T_{\text{el}}/(hf) \ll 1$, these two eigenstates have the same eigenvalue, which is the emission probability $p_1^{(\text{e})} = p_2^{(\text{e})} = 1$. They thus form a degenerated subspace. Any two orthogonal states within this subspace are thus equivalent choices for the two wavefunctions $\varphi_1^{(\text{e})}$ and $\varphi_2^{(\text{e})}$. Indeed, the two-electron state generated at zero temperature is described by a Slater determinant formed from $\varphi_1^{(\text{e})}$ and $\varphi_2^{(\text{e})}$. However, any two orthogonal states within the subspace generated by $\varphi_1^{(\text{e})}$ and $\varphi_2^{(\text{e})}$ would lead to the same Slater determinant. The choice of these two states is thus completely free. This subspace has a dimension 2 and is thus analogous to the Hilbert space of spin $1/2$ which eigenvectors can be mapped to a Bloch sphere. This Bloch sphere representation is plotted on Fig.6. In this representation, we label by $\varphi_{\beta,\pm}$ with $\beta = x, y, z$ the two eigenstates of the spin σ_β, and we set the two wavefunctions, $\varphi_1^{(\text{e})}$ and $\varphi_2^{(\text{e})}$, that we extract from our algorithm, to correspond

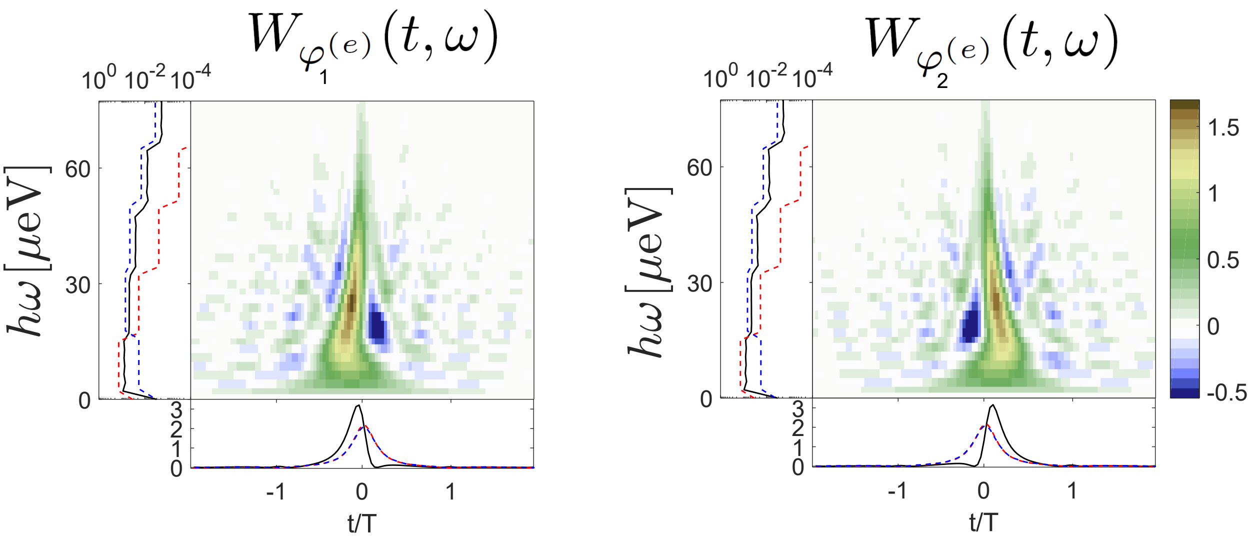

SUPP. FIG. 5. $\varphi_1^{(e)}$ and $\varphi_2^{(e)}$ extracted from $W(t,\omega)$. The red and blue dashed lines represent the Lorentzian wavefunctions $\varphi_{L,n=1}$ and $\varphi_{L,n=2}$.

to the eigenstates of the spin along the y axis, $\varphi_{y,+}$ and $\varphi_{y,-}$. Then, the eigenstates $\varphi_{z,\pm}$ defined as:

$$\varphi_{z,+} = \cos{(\frac{\pi}{4})}e^{i\frac{\pi}{2}}\ \varphi_{y,+} + \sin{(\frac{\pi}{4})}e^{-i\frac{\pi}{2}}\ \varphi_{y,-} \tag{8}$$

$$\varphi_{z,-} = \sin{(\frac{\pi}{4})}e^{i\frac{\pi}{2}}\ \varphi_{y,+} - \cos{(\frac{\pi}{4})}e^{-i\frac{\pi}{2}}\ \varphi_{y,-} \tag{9}$$

correspond to the expected Lorentzian wavefunctions $\varphi_{L,n=1}$ and $\varphi_{L,n=2}$ (see Fig.6). We therefore recover from numerical simulations that at zero temperature, the two particle state is a Slater determinant formed from $\varphi_{L,n=1}$ and $\varphi_{L,n=2}$.

When temperature is increased, $\varphi_1^{(e)}$ and $\varphi_2^{(e)}$ no longer form a degenerate subspace, as $p_1^{(e)} \neq p_2^{(e)}$. The freedom to choose any two orthogonal states within the subspace is removed and temperature favors the emergence of a specific basis. As shown in the main manuscript, we find that $\varphi_1^{(e)}$ and $\varphi_2^{(e)}$ still belong to the same subspace as they have a very strong overlap (almost 1) with the linear combination of $\varphi_{L,n=1}$ and $\varphi_{L,n=2}$ obtained by a rotation of angle $\theta \approx 0.58$ and $\phi = 0$ (red spot on Fig.6). However, even if we observe that $\varphi_1^{(e)}$ and $\varphi_2^{(e)}$ still belong to the same subspace at $T_{el} = 0.05$ K, the generated state is no longer the Slater determinant formed by $\varphi_{L,n=1}$ and $\varphi_{L,n=2}$ as, with probability $p_3^{(e)} \approx 0.24$, $\varphi_1^{(e)}$ is generated together with a different state, $\varphi_3^{(e)}$.

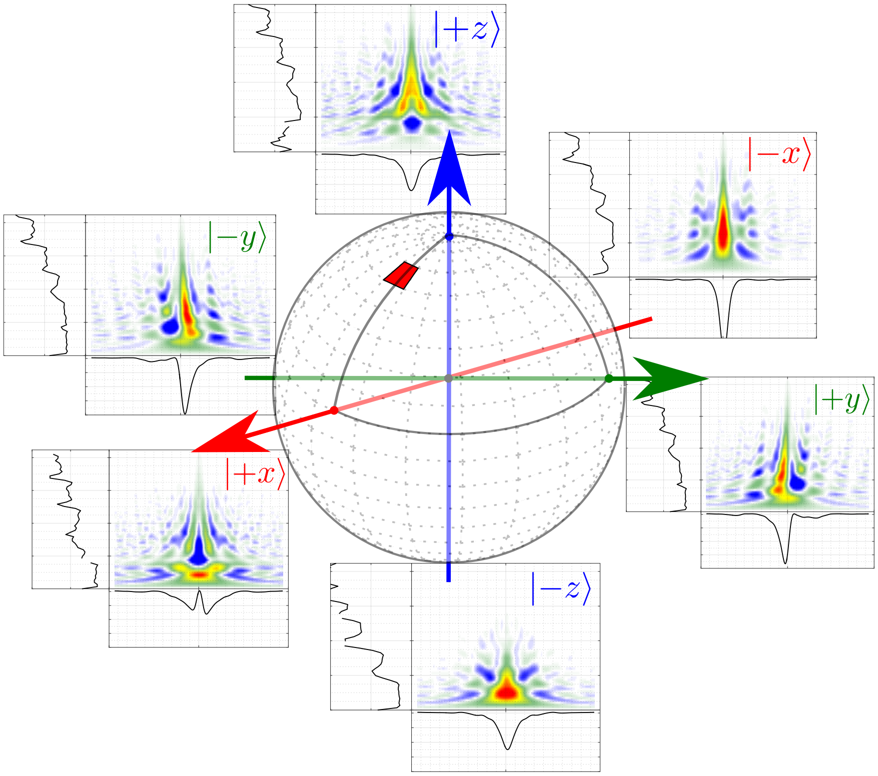

SUPP. FIG. 6. Bloch sphere representation of the two degenerate eigenstates of W projected on the electron sector.

SUPPLEMENTARY NOTE 4. INFLUENCE OF THE DECONVOLUTION PARAMETERS

Our measurements of real and imaginary parts of $\Delta W_{\mathrm{S},n}$ are obtained from noise measurements using deconvolution techniques presented in the Methods section. We present here the influence of the deconvolution parameters on the obtained results on the specific example of the sinusoidal drive at frequency $f = 9$ GHz and temperature $T_{\mathrm{el}} = 60$ mK. More precisely, the deconvolution is performed by adding prior information on the deconvoluted signals using of a Bayesian framework. We assign a gaussian prior distribution on $\mathbf{\Delta W}_{\mathrm{S},n}$:

$$p\left(\mathbf{\Delta W}_{\mathrm{S},n}\middle|\mathbf{V}_f\right) \propto \exp\left(-\frac{1}{2}\left\|\mathbf{\Delta W}_{\mathrm{S},n}\right\|^2_{\mathbf{V}_f}\right). \tag{10}$$

$$V_f(\omega) = v_f \exp\left(-\frac{\omega^2}{w^2}\right), \tag{11}$$

This prior information enforces the physical property that $\Delta W_{\mathrm{S},n}(\omega)$ tends to zero when $|\omega|$ increases on an energy scale set by the parameter w. The parameter v_F is a cut-off parameter for the deconvolution. When v_f is too small, the deconvoluted signals is oversmoothed meaning that, in Fourier space, high frequencies of $\Delta W_{S,n}(\omega)$ which contain relevant information on the physical

signal have been suppressed by the deconvolution filter and some information is lost. This can be detected by reapplying the convolution on the deconvoluted signal; oversmoothing then shows up as a result which differs from the experimentally measured data $\widetilde{\Delta W}_{\mathrm{S},n}$. On the contrary, when v_f is too large, the high frequency values of $\widetilde{\Delta W}_{\mathrm{S},n}$ which are in fact dominated by the noise are amplified by the deconvolution filter which results in the appearance of high frequency fluctuations in the deconvoluted signal. The proper choice of v_f is thus obtained between these two limiting regimes. We plot on Figure 7 the results of the deconvolution algorithm for several values of the parameter V_f varying on one order of magnitude around our estimate of the proper choice for v_f. We can see on the figure that within this range, the results are compatible with each others within error bars, showing the robustness of our deconvolution algorithm to the choice of the input parameter v_f.

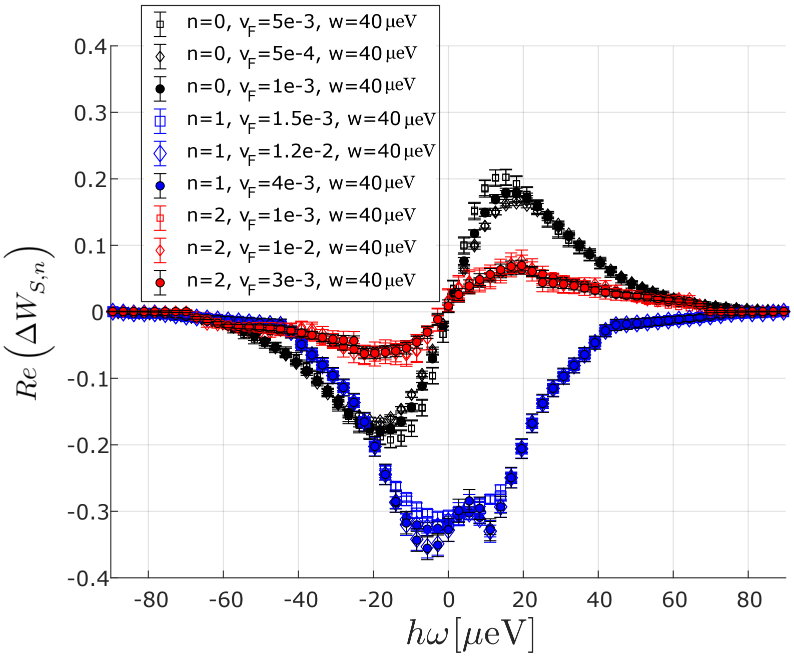

SUPP. FIG. 7. $\Delta W_{\mathrm{S},n}(\omega)$ for the sinusoidal drive at $f = 9$ GHz and $T_{\mathrm{el}} = 60$ mK for different values of the deconvolution parameter v_f.

We next explore on Figure8 the dependence of the deconvolution results on the parameter w which is varied between $w = 20$ μeV and $w = 60$ μeV. The obtained results for $w = 40$ μeV and $w = 60$ μeV are identical. On the contrary, for $w = 20$ μeV, the results are affected by the initial choice of w which enforces $\Delta W_{S,n}(\omega)$ to go to zero on a too small energy scale. We thus choose the value $w = 40$ μeV for all the deconvolutions.

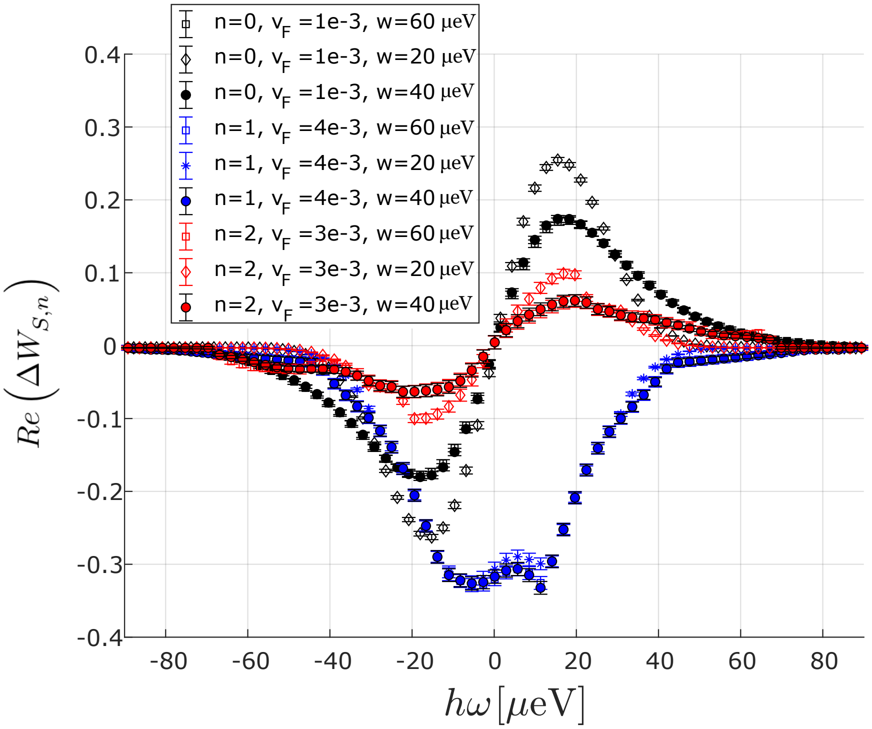

SUPP. FIG. 8. $\Delta W_{\mathrm{S},n}\,(\omega)$ for the sinusoidal drive at $f = 9$ GHz and $T_{\mathrm{el}} = 60$ mK for different values of the deconvolution parameter w.

SUPPLEMENTARY NOTE 5. CALCULATION OF THE ERROR BARS OF $\Delta W_{\mathrm{S},n}\,(\omega)$

We estimate the error bars on $\Delta W_{\mathrm{S},n}\,(\omega)$ using two different methods. The first one relies on a direct calculation derived from the Bayesian framework (see Methods). In this framework, $\Delta W_{\mathrm{S},n}\,(\omega)$ is found by minimizing the following criterion:

$$J\left(\mathbf{\Delta W}_{\mathrm{S},n}\right) = \frac{1}{2}\left\|\widetilde{\mathbf{\Delta W}}_{\mathrm{S},n} - \mathbf{H}_n.\mathbf{\Delta W}_{\mathrm{S},n}\right\|^2_{\mathbf{V}_e} + \frac{1}{2}\left\|\mathbf{\Delta W}_{\mathrm{S},n}\right\|^2_{\mathbf{V}_f}, \tag{12}$$

which results in:

$$\mathbf{\Delta W}_{\mathrm{S},n} = \left(\mathbf{H}^\top \mathbf{V}_e^{-1}\mathbf{H} + \mathbf{V}_f^{-1}\right)^{-1}\mathbf{H}^\top \mathbf{V}_e^{-1}\widetilde{\mathbf{\Delta W}}_{\mathrm{S},n} \tag{13}$$

The error bars are then deduced from the computation of the covariance matrix $\mathbf{C} = \langle \mathbf{\Delta W}_{\mathrm{S},n}\mathbf{\Delta W}_{\mathrm{S},n}{}^\top\rangle - \langle \mathbf{\Delta W}_{\mathrm{S},n}\rangle\langle \mathbf{\Delta W}_{\mathrm{S},n}{}^\top\rangle$. The diagonal elements of $\mathbf{C}$ correspond to the fluctuations (or error bars) of $\langle \Delta W_{\mathrm{S},n}(\omega)^2$ whereas the off-diagonal elements define the correlations of the fluctuations between two different values of ω which result from the deconvolution process. Using Eq.(13) and the definition of the measurement noise $V_e(\omega) = \langle \widetilde{\Delta W}_{\mathrm{S},n}(\omega)^2\rangle - \langle \widetilde{\Delta W}_{\mathrm{S},n}(\omega)\rangle^2$, we directly obtain:

$$\langle C\rangle = \left(\mathbf{H}^{\top}\mathbf{V}_e^{-1}\mathbf{H}+\mathbf{V}_f^{-1}\right)^{-1}\mathbf{H}^{\top}\mathbf{V}_e^{-1}\mathbf{H}\left(\mathbf{H}^{\top}\mathbf{V}_e^{-1}\mathbf{H}+\mathbf{V}_f^{-1}\right)^{-1} \tag{14}$$

The error bars on the deconvoluted Wigner distributions presented in the manuscript are calculated using Eq.(14). The second method allows us to calculate the amplitude of the error bars by generating randomly a large number of noise data $\widetilde{\Delta W}_{\mathrm{S},n}(\omega)$. This random set of data is generated using a Gaussian law which mean value corresponds to our measurement data and with a width given by the measurement noise $V_e(\omega)$. Figure 9 presents the comparison between the estimations of the covariance matrix from these two techniques in the case of the $n = 0$ harmonic of the sinusoidal drive at $f = 9$ GHz and $T_{\mathrm{el}} = 60$ mK. As can be seen on the figure, the two methods for the evaluation of the error bars give exactly the same results.

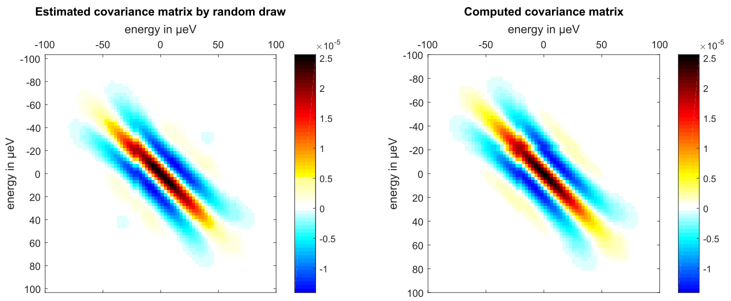

SUPP. FIG. 9. Left, covariance matrix computed by generating randomly sets of noise data. Right, covariance matric computed using Eq.(14).

[1] Ch. Grenier, R. Hervé, E. Bocquillon, F. D. Parmentier, B. Plaçais, J. M. Berroir, G. Fève, and P. Degiovanni, Single-electron quantum tomography in quantum Hall edge channels, *New Journal of Physics* **13**, 093007 (2011).
